# Thyroid Function and Glycemic Profile in Adults with Abnormal Thyroid Function Tests and a Euthyroid Comparison Group: A Cross-Sectional Study from Jazan, Saudi Arabia

**DOI:** 10.3390/medicina62101853

**Published:** 2026-09-24

**Authors:** Yahia A. Mjery, Saif Elden B. Abdalla, Majed A. Suwaydi, Nouf A. Madkhali, Hajar H. Arishi, Ali Baity, Mohammed A. Hassani, Amro Duhduh, Hazem Mathkour, Edrous Alamer, Jobran M. Moshi, Fahd Alaajam, Mohamed M. Almaki, Sarra Kamal Mustafa, Faisal Madkhali, Sultan Mashnafi, Hassan Mari

**Affiliations:** 1 Department of Medical Laboratory Technology, College of Nursing and Health Sciences, Jazan University, Jazan 45142, Saudi Arabia; sebrahim@jazanu.edu.sa (S.E.B.A.); msuwaydi@jazanu.edu.sa (M.A.S.); noufabmad@gmail.com (N.A.M.); hr.h75@hotmail.com (H.H.A.); abaity@jazanu.edu.sa (A.B.); mhassani@jazanu.edu.sa (M.A.H.); aduhduh@jazanu.edu.sa (A.D.); hmathkour@jazanu.edu.sa (H.M.); ealamer@jazanu.edu.sa (E.A.); jmoshi@jazanu.edu.sa (J.M.M.); falaajam@jazanu.edu.sa (F.A.); malmaki@jazanu.edu.sa (M.M.A.); skamal@jazanu.edu.sa (S.K.M.); fmadkhali@jazanu.edu.sa (F.M.); 2 Graduate Studies and Scientific Research Department, Jazan University Hospital, Jazan University, Jazan 45142, Saudi Arabia; 3 Department of Basic Medical Sciences, Faculty of Applied Medical Sciences, Al-Baha University, Al-Baha 65431, Saudi Arabia; sqasem@bu.edu.sa; 4 Laboratory Department, Baish General Hospital, Jazan 87389, Saudi Arabia; almriee@hotmail.com

**Keywords:** thyroid dysfunction, hypothyroidism, hyperthyroidism, glycated hemoglobin, fasting plasma glucose, thyrotropin, cross-sectional study, spectrum bias, Saudi Arabia

## Abstract

*Background and Objectives:* Thyroid dysfunction and dysglycemia often coexist, but cross-sectional associations may reflect the populations sampled rather than a graded biochemical relationship. We compared the glycemic profile of adults with abnormal thyroid function tests with that of a euthyroid comparison group and examined whether thyrotropin (TSH), free thyroxine (FT4) and free triiodothyronine (FT3) were associated with glycated hemoglobin (HbA1c) and fasting plasma glucose (FPG) within groups. *Materials and Methods:* This cross-sectional study included 331 adults from Jazan, Saudi Arabia: 230 with abnormal thyroid function tests from three hospital laboratories and 101 patients selected for normal thyroid function tests and normal glucose values. Participants were classified by TSH as euthyroid (*n* = 102), hyperthyroid pattern (*n* = 56) or hypothyroid pattern (*n* = 173). Spearman correlations, Kruskal–Wallis tests and robust linear regression were used, with multiple-imputation, threshold, restriction and sex-stratified sensitivity analyses. *Results:* In the whole cohort, TSH correlated with HbA1c (ρ = 0.59) and FPG (ρ = 0.63; both *p* < 0.001); median HbA1c was 5.1%, 5.8% and 6.6% in the euthyroid, hyperthyroid pattern and hypothyroid pattern groups. Within the hypothyroid pattern group, TSH was unrelated to HbA1c (ρ = 0.00) and FPG (ρ = 0.07), and overt and subclinical patterns had the same median HbA1c (6.6%). Thyroid function group alone explained 46% of HbA1c variance; continuous thyroid parameters, age, sex and body mass index explained 18%. *Conclusions:* The between-group differences and pooled correlations reflect a two-source design in which the comparison group was selected for normal glucose values. No graded relationship between thyroid function and glycemia was detectable within groups, and causal or directional inference is not supported.

## 1. Introduction

Thyroid disorders and diabetes mellitus are among the most prevalent endocrine conditions globally, collectively affecting hundreds of millions of individuals, and their frequent coexistence has led to their description as twin epidemics [1,2,3,4,5]. The burden of both conditions is substantial in Saudi Arabia, where thyroid dysfunction has been detected in a large proportion of adults undergoing thyroid testing in the southern region [6] and in roughly one-quarter to one-third of patients with diabetes [7,8]. The thyroid gland regulates basal metabolic rate, thermogenesis, and glucose homeostasis through the actions of its two hormones, thyroxine (T4) and triiodothyronine (T3), under the control of pituitary thyroid-stimulating hormone (TSH) [9,10,11].

Experimental studies have characterized distinct mechanisms linking thyroid dysfunction to glycemic changes. Hypothyroidism is associated in cell and animal models with reduced insulin-mediated glucose uptake, impaired glycogenolysis, and decreased peripheral glucose oxidation [9,12,13,14]. Conversely, hyperthyroidism accelerates hepatic glucose production in vitro, increases intestinal glucose absorption, and potentiates insulin resistance through catecholamine excess and mitochondrial uncoupling [15,16]. Whether these mechanisms operate in vivo in humans with naturally occurring thyroid dysfunction remains incompletely understood.

Glycated hemoglobin (HbA1c) is the gold-standard biomarker for long-term glycemic control and diabetes diagnosis [17,18]. Thyroid dysfunction may affect HbA1c interpretation, as alterations in erythrocyte turnover, hemoglobin glycation kinetics, and red-cell lifespan associated with thyroid function can independently influence HbA1c values [19,20,21]. Several cross-sectional observational studies have reported positive correlations between elevated TSH and HbA1c [22,23,24], and meta-analyses of prospective cohorts have linked higher TSH and lower free thyroid hormone concentrations with incident type 2 diabetes [25,26], raising the hypothesis that thyroid dysfunction and glycemic control may be related. However, an individual-participant-data analysis of 18 cohorts found no association between subclinical thyroid dysfunction and incident diabetes [27], cross-sectional associations are inherently bidirectional, and the relative contribution of causality versus confounding remains unclear [28,29].

Epidemiological evidence indicates that thyroid autoimmune disease and type 2 diabetes mellitus frequently co-occur, with shared immunological and metabolic risk factors including obesity, insulin resistance, and chronic low-grade inflammation [30,31,32,33,34,35]. Higher body mass index (BMI) has been associated with modestly higher TSH concentrations within the reference range in population-based studies [36,37], although the great majority of people with obesity have normal thyroid function; BMI is therefore a potential confounder of thyroid–glycemia associations rather than a determinant of TSH. In addition, common medications such as metformin, which lowers TSH in hypothyroid patients [38], and glucocorticoids, which suppress TSH [39], and comorbidities affect both thyroid function and glycemic control, and these potential confounders should be borne in mind when interpreting cross-sectional associations.

A further, often overlooked, determinant of the size of a cross-sectional correlation is the way the sample was assembled. When a dataset combines patients selected because of an abnormal test result with a separately assembled group of healthy individuals, differences between the two sources in any characteristic that also differs between the groups inflate correlations across the pooled sample, a form of spectrum bias closely related to the selection effect described by Berkson [40,41]. Such correlations may be large even when no graded relationship exists within either group. Examining associations within groups is therefore essential before a pooled correlation is interpreted biologically.

Most existing studies of thyroid–glucose relationships have examined single thyroid parameters or have lacked adjustment for BMI, sex, and age [22,23,24], and few have tested whether the associations they report persist within thyroid function categories. The present study aimed to (1) describe the distribution of thyroid function parameters and glycemic markers by thyroid function category in adults with abnormal thyroid function tests and in a euthyroid comparison group; (2) quantify correlations between TSH, FT4, FT3, HbA1c, and fasting plasma glucose (FPG) in the whole cohort and within each thyroid function group; (3) compare glycemic-marker distributions across thyroid function groups; and (4) examine associations of thyroid parameters with glycemic markers in multivariable regression models adjusted for age, sex and BMI.

## 2. Materials and Methods

### 2.1. Study Design

This was a cross-sectional analysis of de-identified laboratory and anthropometric records assembled from two sources in the Jazan region of southwestern Saudi Arabia. The study is reported in accordance with the STROBE recommendations for cross-sectional studies. The numbers of participants at each stage are given in Section 3.1 and Figure 1.

### 2.2. Study Population, Data Sources and Eligibility Criteria

Two groups of records were assembled. The abnormal thyroid function group comprised records of adults whose thyroid function tests showed at least one result outside the laboratory reference interval, retrieved from the laboratories of three Ministry of Health hospitals in the Jazan region: King Fahd Central Hospital, Sametah General Hospital, and Baish General Hospital. The euthyroid comparison group comprised 101 patients attending King Fahd Central Hospital for routine laboratory investigations who were selected because their TSH, FT4 and FT3 and their glucose values were all within the reference intervals; they were added to bring the dataset toward a planned target of 400 records, and the final dataset comprised 331 records. The site field was not completed in the extracted dataset for 100 of these participants and reads Sametah General Hospital for one. Records were retrieved for the period March 2025 to March 2026, the period covered by the ethics approval. Because the two groups were assembled independently, and because the comparison group was selected for normal glucose values as well as normal thyroid function, the cohort is a two-source clinical laboratory sample rather than a consecutive series of unselected patients; by construction, the comparison group has lower glycemic values than the patient group, and this is taken into account in the analysis and interpretation.

Inclusion criteria were age 18 years or older and availability of TSH, FT4 and FT3 measured on the same occasion together with age, sex, body weight and height. Exclusion criteria were pregnancy, known hematological disorders affecting red blood cell lifespan, and acute illness at the time of sampling, as documented in the laboratory request; records that failed these criteria were not entered into the dataset. HbA1c and FPG were retrieved when available and their availability is reported in Section 3.3. Clinical diagnoses (including diabetes, thyroid disease, pituitary disease and thyroid autoimmunity), current medication (including levothyroxine, antithyroid drugs, glucose-lowering drugs, glucocorticoids and other drugs affecting thyroid function), thyroid antibody status and pituitary imaging were not available in the extracted laboratory records. Consequently, treated and untreated thyroid dysfunction could not be distinguished, participants with a prior diagnosis of diabetes or thyroid disease could not be identified, and pituitary causes of an elevated TSH (for example a TSH-secreting adenoma) or autoimmune etiology could not be excluded. These constraints are addressed in the sensitivity analyses (Section 2.7) and in Section 4.8.

### 2.3. Laboratory Measurements

TSH, FT4 and FT3 were measured by paramagnetic-particle chemiluminescent immunoassay (CLIA) using Access thyroid assays on a Beckman Coulter Access immunoassay system (Beckman Coulter, Brea, CA, USA). The manufacturer’s adult reference intervals were TSH 0.34–5.60 mIU/L, FT4 7.86–14.41 pmol/L and FT3 3.80–6.00 pmol/L. Intra-assay and inter-assay coefficients of variation were <5%. Values at the limits of the analytical measuring range were retained as reported (TSH at the lower measuring limit in 12 records and FT3 at the upper measuring limit of 30.7 pmol/L in 7 records); their influence is addressed in Section 2.7.

HbA1c was measured in EDTA-anticoagulated whole blood by a turbidimetric inhibition immunoassay (HbA1c Advanced; Beckman Coulter) on a DxC 700 AU clinical chemistry analyzer (Beckman Coulter, Brea, CA, USA); the assay is certified by the National Glycohemoglobin Standardization Program (NGSP) and traceable to the International Federation of Clinical Chemistry and Laboratory Medicine (IFCC) reference method. FPG was measured in plasma by the hexokinase method on the same DxC 700 AU analyzer; normal fasting glucose was defined as <5.6 mmol/L in accordance with American Diabetes Association criteria [18].

Laboratory results were retrieved retrospectively from the medical records. FPG values were analyzed only when overnight fasting (≥8 h) and morning blood collection (06:00–09:00) were documented in the record; thyroid function tests and HbA1c, which do not require fasting, were retrieved irrespective of fasting status. All assays were conducted in ISO 15189-accredited laboratories implementing routine internal quality control (two levels daily), monthly calibration checks, and external quality assurance (EQAS/CAP).

### 2.4. Anthropometric Measurements

Body weight and height were measured using standardized protocols, and BMI was calculated as weight (kg) divided by height squared (m^2^). Two height values incompatible with life (100 and 107 cm) were treated as missing, so BMI was available for 329 participants.

### 2.5. Thyroid Function Categories

Participants were classified into three thyroid function categories according to serum TSH, the primary screening test recommended by current guidelines [1,42,43]: euthyroid, TSH 0.4–4.0 mIU/L; hyperthyroid pattern, TSH <0.4 mIU/L; and hypothyroid pattern, TSH > 4.0 mIU/L. These prespecified thresholds correspond to the upper reference limit of approximately 4.0 mIU/L recommended in clinical guidelines [1,42,43] and were applied uniformly to all records. The 4.0 mIU/L cut-off was chosen a priori because it is the guideline-endorsed population upper limit used in most comparable studies [22,23,24], whereas the manufacturer’s upper limit of 5.60 mIU/L is an assay-specific reference interval; participants with TSH between 4.0 and 5.6 mIU/L were therefore assigned to the hypothyroid pattern group. The consequences of this choice were examined by reclassifying participants at 4.5 and 5.6 mIU/L (Section 2.7). FT4 and FT3 were not used as classification criteria; they were analyzed as continuous variables and were used descriptively to subdivide the hypothyroid pattern into overt (FT4 below 7.86 pmol/L) and subclinical (FT4 within the reference interval) biochemical patterns, and the hyperthyroid pattern into overt (FT4 above 14.41 pmol/L or FT3 above 6.00 pmol/L) and subclinical patterns. The terms “hyperthyroid pattern” and “hypothyroid pattern” denote single-point biochemical patterns at the time of testing rather than confirmed clinical diagnoses, because treatment status, repeat measurements and thyroid antibody data were not available.

### 2.6. Glycemic Categories

HbA1c was categorized as normal (<5.7%), prediabetes range (5.7–6.4%), or diabetes range (≥6.5%) [17,18]. FPG categories were defined as normal (<5.6 mmol/L), impaired fasting glucose (5.6–6.9 mmol/L), and diabetes range (≥7.0 mmol/L). These categories describe single laboratory values and do not constitute clinical diagnoses of prediabetes or diabetes.

### 2.7. Statistical Analysis

Statistical analyses were performed in Python 3.12 using SciPy 1.17 [44] and statsmodels 0.15 [45]. Continuous variables were assessed for normality using the Shapiro–Wilk test. Because all major biochemical variables showed non-normal distributions, descriptive statistics are presented as median [interquartile range, IQR], and non-parametric methods were used for group comparisons and correlation analyses.

*Descriptive analyses.* Group differences in continuous variables across thyroid function groups or glycemic categories were evaluated using the Kruskal–Wallis test, with Dunn’s post hoc test and Bonferroni correction for pairwise comparisons [46]. Bivariate associations were assessed using Spearman rank correlation, with 95% confidence intervals (CIs) obtained by bootstrap resampling (2000 resamples); Pearson and Kendall coefficients were computed as alternative measures (Supplementary Table S7). Correlations were computed in the whole cohort and separately within each thyroid function group, because a correlation across a two-source sample can be generated entirely by differences between the sources. The association between thyroid function category and HbA1c category was assessed using Pearson’s chi-square test with Cramér’s V as the effect size.

*Regression models.* Because TSH was right-skewed over five orders of magnitude, it was natural-log transformed (ln TSH) in the continuous models; a regression coefficient for ln TSH multiplied by ln 2 (0.693) gives the change in outcome per doubling of TSH. Three sets of ordinary least-squares models were fitted: (A) group-indicator models, HbA1c or FPG regressed on thyroid function group (reference: euthyroid), age, sex and BMI; (B) continuous models, HbA1c or FPG regressed on ln TSH, FT4, FT3, age, sex and BMI in the whole cohort; and (C) the continuous models fitted within the hypothyroid pattern group alone (ln TSH, FT4, age, sex and BMI), which test for a graded relationship among participants who all have an elevated TSH. A model with ln TSH as the outcome and HbA1c and FPG as predictors was fitted only as a symmetry check: reversing outcome and predictors in cross-sectional data yields mathematically related estimates and cannot demonstrate bidirectional biological effects (Supplementary Table S8). All coefficients are reported as unstandardized β with heteroscedasticity-consistent (HC3) standard errors and 95% CI [47], together with standardized coefficients (per standard deviation); model-based and bootstrap intervals are given in Supplementary Table S8. A clinical diagnosis of diabetes was not available and could not be entered as a covariate; instead, analyses restricted by laboratory-defined glycemic range are reported as sensitivity analyses. Adjusted R^2^ and omnibus F-statistics are reported. Statistical significance was set at α = 0.05.

*Model diagnostics.* Regression assumptions were assessed by inspection of residual-versus-fitted plots (linearity and homoscedasticity), normal quantile–quantile (Q–Q) plots of standardized residuals (normality), the Breusch–Pagan test, variance inflation factors (VIFs; multicollinearity), and Cook’s distance (influential observations); each model was re-estimated after excluding observations with Cook’s distance greater than 4/*n*.

*Sensitivity analyses.* Four prespecified sensitivity analyses were performed. (i) Threshold: participants were reclassified using upper TSH limits of 4.5 and 5.60 mIU/L (the manufacturer’s limit) and a lower limit of 0.34 mIU/L, and group sizes, prevalence estimates and group comparisons were recomputed. (ii) Missing data: multiple imputation by chained equations with predictive mean matching (m = 20 imputed datasets, 10 iterations each) were used to impute HbA1c and FPG from ln TSH, FT4, FT3, age, sex, BMI, thyroid function group and site; correlations (after Fisher transformation) and regression coefficients were pooled using Rubin’s rules [48,49,50]. (iii) Restriction: analyses were repeated after excluding participants with any diabetes range value (HbA1c ≥ 6.5% or FPG ≥ 7.0 mmol/L) and, separately, in participants with values in the normoglycemic range only (HbA1c < 5.7% and FPG < 5.6 mmol/L). (iv) Sex: correlations were computed separately in women and men, among euthyroid and hypothyroid pattern participants (the two larger groups) and in the whole cohort, with differences tested by Fisher’s z, and a ln TSH by sex interaction term was added to the continuous models.

### 2.8. Handling of Missing Data

Bivariate and regression analyses used pairwise and complete-case data, respectively, and the pattern of missingness was characterized by thyroid function group and site and by logistic regression of missingness on group, age, sex and BMI. Because the extent of missing glycemic data differed markedly between groups, the multiple-imputation analysis described in Section 2.7 was used to assess the sensitivity of the main estimates to the missing-at-random assumption.

### 2.9. Sensitivity Power Analysis

A target of 400 records was set at the planning stage and 331 were obtained. A sensitivity power analysis was therefore performed to estimate the minimum effect size detectable with the available sample. For the primary comparison of HbA1c across thyroid function groups, the available HbA1c sample (*n* = 294) provided 80% power at α = 0.05 to detect a Cohen’s *f* of approximately 0.18. For correlation analyses, the available sample sizes provided 80% power to detect correlations of approximately r = 0.16 (HbA1c) and r = 0.17 (FPG); within the hypothyroid pattern group (*n* = 163 with HbA1c), a correlation of approximately r = 0.22 was detectable.

### 2.10. Ethical Considerations

The study protocol was approved by the Local Committee for Research Ethics, Jazan University (HAPO-10-Z-001; Reference No.: REC-46/09/1407, decision dated 4 March 2025), access to the hospital laboratory records was granted under the research agreement between Jazan University and the Ministry of Health (Jazan Health Cluster), and the requirement for individual informed consent was waived owing to the retrospective analysis of de-identified records.

### 2.11. Use of Generative Artificial Intelligence

Generative AI (Claude, Anthropic, San Francisco, CA, USA) was used under the authors’ direction as a tool for language editing, template formatting and the preparation of analysis scripts and figures. All analyses, results and interpretations were checked and approved by the authors, who take full responsibility for the content of the work.

## 3. Results

### 3.1. Sample Characteristics

The study dataset comprised 331 adult participants (199 women, 60.1%; 132 men, 39.9%) with a median age of 37 [IQR 31–43] years (range 18–85): 230 from the abnormal thyroid function group (King Fahd Central Hospital, *n* = 100; Sametah General Hospital, *n* = 99; Baish General Hospital, *n* = 31) and 101 from the euthyroid comparison group recruited at King Fahd Central Hospital (Figure 1). By TSH, 173 participants (52.3%) had a hypothyroid pattern, 102 (30.8%) were euthyroid and 56 (16.9%) had a hyperthyroid pattern. Four participants from the euthyroid comparison group (TSH 4.1–4.5 mIU/L) fell into the hypothyroid pattern group and five from the abnormal thyroid function group with abnormal FT4 or FT3 but normal TSH fell into the euthyroid group. Within the hypothyroid pattern, 86 participants had a low FT4 (overt pattern) and 87 a normal FT4 (subclinical pattern); within the hyperthyroid pattern, 54 had an elevated FT4 or FT3 and 2 did not. The median BMI was 25.39 [IQR 22.50–29.41] kg/m^2^. Characteristics overall and by thyroid function group are presented in Table 1; the groups did not differ in age, but euthyroid comparison participants had a markedly lower BMI than either abnormal-thyroid group.

### 3.2. Laboratory-Defined Glycemic Categories

Among the 294 participants with HbA1c, 121 (41.2%) had a normal value, 70 (23.8%) a prediabetes range value and 103 (35.0%) a diabetes range value. Among the 269 participants with FPG, 104 (38.7%) had a normal value, 139 (51.7%) impaired fasting glucose and 26 (9.7%) a diabetes range value. Any diabetes range value on either marker was present in 107 participants (32.3%). In the abnormal thyroid function group, 88.1% of participants with HbA1c had a value of 5.7% or higher (52.8% at 6.5% or higher), compared with 3.0% in the euthyroid comparison group. Documented clinical diagnoses and treatment status were not available in the records.

### 3.3. Missing Data

HbA1c was available for 294 participants (88.8%; 37 missing, 11.2%) and FPG for 269 (81.3%; 62 missing, 18.7%); both markers were available for 268. Missingness was concentrated in the hyperthyroid pattern group, in which HbA1c was missing in 25 of 56 (44.6%) and FPG in 47 of 56 (83.9%), compared with a respective 10 of 173 (5.8%) and 11 of 173 (6.4%) in the hypothyroid pattern group and 2 of 102 and 4 of 102 in the euthyroid group. All missing glycemic values occurred in the abnormal thyroid function group at two sites: King Fahd Central Hospital (HbA1c missing in 27 and FPG in 38 of 100 records) and Sametah General Hospital (HbA1c missing in 10 and FPG in 24 of 99 records); no values were missing at Baish General Hospital or in the euthyroid comparison group. In logistic regression, the hyperthyroid pattern was strongly associated with missing HbA1c (odds ratio 48.0 versus euthyroid, *p* < 0.001), whereas age, sex and BMI were not. Within the hyperthyroid pattern group, participants with and without HbA1c did not differ significantly in age, sex, BMI, TSH, FT4 or FT3 (all *p* > 0.10; Supplementary Table S2). Missingness therefore depended on group and site rather than on measured participant characteristics, a pattern compatible with data missing at random conditional on group, although missingness related to unmeasured clinical factors cannot be excluded.

### 3.4. Distribution Assessment

Shapiro–Wilk testing showed significant deviation from normality for all continuous variables (all *p* ≤ 0.01). Continuous variables are therefore presented as median [IQR], and non-parametric methods were used for group comparisons and correlation analyses.

### 3.5. Correlation Analysis in the Whole Cohort

In the whole cohort, TSH showed positive correlations with HbA1c (ρ = 0.59, 95% CI 0.52 to 0.65, *n* = 294) and FPG (ρ = 0.63, 95% CI 0.55 to 0.70, *n* = 269) (both *p* < 0.001). FT4 and FT3 showed inverse correlations with HbA1c (ρ = −0.24 and ρ = −0.38, respectively; both *p* < 0.001), and BMI correlated positively with HbA1c (ρ = 0.41), FPG (ρ = 0.48) and TSH (ρ = 0.28; all *p* < 0.001). The full set of bivariate correlations is presented in Table 2 and summarized in Figure 2; Pearson coefficients, which are sensitive to the extreme TSH, FT4 and FT3 values, were consistently smaller than the rank-based coefficients for pairs involving thyroid hormones (Table 2). Scatter plots showing each participant are given in Figure 3.

### 3.6. Correlation Analysis Within Thyroid Function Groups

The pooled correlations were not reproduced within groups (Table 3; Figure 3). Within the hypothyroid pattern group, TSH was not correlated with HbA1c (ρ = 0.00 (95% CI −0.16 to 0.15), *n* = 163, *p* = 0.99) or FPG (ρ = 0.07 (95% CI −0.09 to 0.24), *n* = 162, *p* = 0.39), and neither FT4 nor FT3 was correlated with either glycemic marker. Within the euthyroid group, TSH was likewise unrelated to HbA1c (ρ = 0.08, *p* = 0.43) and FPG (ρ = 0.09, *p* = 0.37). In the smaller hyperthyroid pattern group, FT4 was inversely correlated with HbA1c (ρ = −0.41, *n* = 31, *p* = 0.022), whereas TSH was not (ρ = 0.24, *p* = 0.19); only 9 hyperthyroid pattern participants had FPG. Within the abnormal thyroid function group as a whole (hyperthyroid and hypothyroid patterns combined), TSH correlated modestly with HbA1c (ρ = 0.24, *p* < 0.001), reflecting the lower HbA1c of the hyperthyroid pattern subgroup, but not with FPG (ρ = 0.02, *p* = 0.75). BMI was not correlated with HbA1c or FPG within any group. The expected inverse TSH–FT4 relationship was present within both abnormal groups (hypothyroid pattern ρ = −0.44; hyperthyroid pattern ρ = −0.53; both *p* < 0.001), confirming that the absence of within-group thyroid–glycemia correlations was not due to a lack of variation in the thyroid parameters.

### 3.7. Group Comparisons

Kruskal–Wallis testing demonstrated significant differences in HbA1c across thyroid function groups (H = 182.86, df = 2, *p* < 0.001; ε^2^ = 0.62). Median HbA1c was 5.10% [4.90–5.20] in the euthyroid group, 5.80% [5.35–5.95] in the hyperthyroid pattern group, and 6.60% [5.90–7.30] in the hypothyroid pattern group (Figure 4A); all pairwise differences were significant after Bonferroni correction (euthyroid versus hypothyroid pattern *p* < 0.001; euthyroid versus hyperthyroid pattern *p* < 0.001; hyperthyroid versus hypothyroid pattern *p* < 0.001). Within the hypothyroid pattern, median HbA1c was identical in the overt (6.6% [5.9–7.3], *n* = 78) and subclinical (6.6% [6.0–7.3], *n* = 85) sub-patterns (Mann–Whitney *p* = 0.73), as was median FPG (6.1 versus 6.1 mmol/L; *p* = 0.80; Supplementary Table S1). Because the comparison group was selected for normal glucose values (Section 2.2), the differences between it and the abnormal thyroid groups are reported for description only.

TSH differed significantly across HbA1c categories (H = 116.23, df = 2, *p* < 0.001), with median values of 2.10 [IQR 1.10–2.80] mIU/L in the normal HbA1c category, 9.35 [7.40–12.57] mIU/L in the prediabetes range, and 9.70 [8.40–12.10] mIU/L in the diabetes range (Figure 4B); TSH did not differ between the prediabetes range and diabetes range categories (Bonferroni-adjusted *p* = 0.42). Significant group differences were also observed for FPG (H = 174.61, df = 2, *p* < 0.001; medians 4.5, 6.1 and 6.1 mmol/L; the hyperthyroid pattern estimate rests on 9 participants) and BMI (H = 69.02, df = 2, *p* < 0.001; medians 22.8, 25.8 and 26.8 kg/m^2^) across thyroid function groups, whereas age did not (H = 0.47, *p* = 0.79).

### 3.8. Chi-Square Analysis

The distribution of HbA1c categories differed significantly by thyroid function group (χ^2^ = 215.58, df = 4, *p* < 0.001; Cramér’s V = 0.61). Among euthyroid individuals with HbA1c data, 0 of 100 (0.0%) met diabetes range HbA1c criteria and 97 (97.0%) had a normal value, whereas, among participants with a hypothyroid pattern, 97 of 163 (59.5%) were in the diabetes range and only 11 (6.7%) had a normal value; the corresponding figures in the hyperthyroid pattern group were 6 of 31 (19.4%) and 13 (41.9%). The distribution of FPG categories showed the same pattern (χ^2^ = 239.52, df = 4, *p* < 0.001).

### 3.9. Multivariable Regression Analysis

*Group-indicator models.* Adjusted for age, sex and BMI, the hypothyroid pattern was associated with an HbA1c higher by 1.68 percentage points (95% CI 1.48 to 1.88) and the hyperthyroid pattern by 0.84 percentage points (95% CI 0.51 to 1.17) relative to the euthyroid group (both *p* < 0.001; R^2^ = 0.467, adjusted R^2^ = 0.458, *n* = 293). For FPG the corresponding differences were 1.67 mmol/L (95% CI 1.46 to 1.87) and 1.58 mmol/L (95% CI 1.27 to 1.88) (R^2^ = 0.623, *n* = 268). Thyroid function group alone explained 46.3% of the variance in HbA1c and 61.9% of the variance in FPG, and once the group was in the model BMI was no longer associated with either outcome (HbA1c: β = 0.003, 95% CI −0.017 to 0.022, *p* = 0.800; FPG: β = 0.011, 95% CI −0.007 to 0.030, *p* = 0.239) (Table 4). These coefficients quantify the separation between the groups that was built into the sampling design and should not be read as effects of thyroid function.

*Continuous models in the whole cohort.* ln TSH (β = 0.179, 95% CI 0.076 to 0.282, *p* < 0.001; standardized β = 0.35) and BMI (β = 0.054, 95% CI 0.033 to 0.075, *p* < 0.001; standardized β = 0.27) were associated with HbA1c, whereas FT4, FT3, age and sex were not; the model explained 18.1% of the variance (R^2^ = 0.181; adjusted R^2^ = 0.164; F(6, 286) = 10.52; *p* < 0.001; *n* = 293). The coefficient corresponds to an HbA1c higher by 0.12 percentage points per doubling of TSH and by 0.41 percentage points per tenfold increase, compared with 0.27 percentage points per 5 kg/m^2^ of BMI. For FPG, ln TSH (β = 0.457, 95% CI 0.245 to 0.669, *p* < 0.001; standardized β = 0.63), FT3 (β = 0.184, 95% CI 0.092 to 0.277, *p* < 0.001) and BMI (β = 0.062, 95% CI 0.038 to 0.086, *p* < 0.001) were associated with the outcome and FT4 was not (β = −0.041, 95% CI −0.087 to 0.005, *p* = 0.079); R^2^ = 0.381 (adjusted R^2^ = 0.367; F(6, 261) = 26.78; *p* < 0.001; *n* = 268). Omitting FT3, which is highly collinear with FT4, changed the ln TSH and BMI coefficients by less than 15% (Supplementary Table S8).

*Models within the hypothyroid pattern group.* Among participants with an elevated TSH, none of ln TSH (β = 0.139, 95% CI −0.248 to 0.525, *p* = 0.482), FT4, age, sex or BMI were associated with HbA1c, and the model explained 2.0% of the variance (adjusted R^2^ = −0.011; F test *p* = 0.67; *n* = 162). For FPG, the ln TSH coefficient was positive (β = 0.534, 95% CI −0.076 to 1.144, *p* = 0.086 with HC3 standard errors; model-based *p* < 0.001), but the estimate depended on a single influential observation (maximum Cook’s distance 0.93) and the corresponding rank correlation was negligible (ρ = 0.07); R^2^ = 0.119, *n* = 161. Adjusted regression coefficients for the primary models are displayed in Table 4 and Figure 5, and the symmetry check with ln TSH as the outcome is reported in Supplementary Table S8 (HbA1c and FPG both associated with ln TSH, R^2^ = 0.210), as expected from the pooled correlations.

### 3.10. Regression Model Diagnostics

*Multicollinearity.* Variance inflation factors were below 3 for ln TSH (2.75), age, sex and BMI, but were 7.5 for FT4 and 6.9 for FT3 in the whole-cohort HbA1c model, reflecting their strong biological coupling (Pearson r = 0.93). Because FT4 and FT3 were covariates rather than the exposure of interest, both were retained; omitting FT3 did not materially alter the ln TSH or BMI estimates (Supplementary Table S8).

*Linearity and homoscedasticity.* Residual-versus-fitted plots (Supplementary Figure S1) showed no systematic curvature after log-transformation of TSH; the Breusch–Pagan test did not indicate heteroscedasticity in the ln TSH models (*p* = 0.62 and 0.87), whereas the original specification with untransformed TSH showed heteroscedasticity (*p* = 0.008 and *p* < 0.001) and its TSH coefficients were not significant under HC3 standard errors (Supplementary Table S8). HC3 standard errors are therefore reported throughout.

*Normality of residuals.* Q–Q plots showed departures from normality in both tails (Shapiro–Wilk *p* < 0.001 for all models), consistent with the clustered structure of the data. Bootstrap 95% confidence intervals (1000 resamples) agreed closely with the HC3 intervals (Supplementary Table S8).

*Influential observations.* Maximum Cook’s distances were 0.086 (whole-cohort HbA1c model), 0.265 (whole-cohort FPG model), 0.077 (hypothyroid pattern HbA1c model) and 0.93 (hypothyroid pattern FPG model); 17 and 13 observations exceeded 4/*n* in the two whole-cohort models. Re-estimation after excluding these observations did not change the direction or significance of the ln TSH and BMI coefficients in the whole-cohort models (Supplementary Table S8).

### 3.11. Sensitivity Analyses

*TSH classification threshold.* Raising the upper TSH limit from 4.0 to 4.5 or to the manufacturer’s 5.60 mIU/L reclassified only 4 participants (TSH 4.1–4.5 mIU/L, all from the euthyroid comparison group; median HbA1c 5.1%), because no participant had a TSH between 4.5 and 6.2 mIU/L; the hypothyroid pattern group decreased from 173 (52.3%) to 169 (51.1%), the euthyroid group increased to 106 (32.0%), medians were unchanged, and the Kruskal–Wallis statistic for HbA1c changed from 182.9 to 187.8. Lowering the lower limit to 0.34 mIU/L reclassified no one (Supplementary Table S3). The continuous analyses are unaffected by the threshold.

*Multiple imputation.* After imputation of missing HbA1c and FPG (m = 20), the pooled TSH–HbA1c correlation was 0.54 (95% CI 0.45 to 0.62) compared with 0.59 in the complete-case analysis, whereas the TSH–FPG correlation decreased from 0.63 to 0.37 (95% CI 0.24 to 0.49), reflecting the 83.9% of hyperthyroid pattern participants without FPG. The group-indicator estimates were stable (hypothyroid pattern versus euthyroid: HbA1c 1.67 percentage points, 95% CI 1.44 to 1.90; FPG 1.60 mmol/L, 95% CI 1.42 to 1.79). In the continuous whole-cohort models, the ln TSH coefficient for HbA1c was 0.148 (95% CI 0.069 to 0.227; *p* < 0.001) but the coefficient for FPG was no longer significant (0.081, 95% CI −0.012 to 0.174; *p* = 0.09), while the BMI coefficients were unchanged (Supplementary Table S4). The continuous TSH–FPG association is therefore sensitive to the assumption made about the missing FPG values of hyperthyroid pattern participants.

*Restriction by glycemic range.* After excluding the 107 participants (32.3%) with any diabetes range value (*n* = 224), the TSH–HbA1c correlation decreased from 0.59 to 0.44 (95% CI 0.30 to 0.57) and the TSH–FPG correlation from 0.63 to 0.61; in the continuous models the ln TSH coefficient for HbA1c was no longer significant (0.035, 95% CI −0.027 to 0.097; *p* = 0.27) whereas the coefficient for FPG remained significant (0.391, 95% CI 0.177 to 0.604; *p* < 0.001). Restriction to the normoglycemic range retained 113 participants, of whom only 3 had a hypothyroid pattern and 13 a hyperthyroid pattern, so that this subset was almost entirely the euthyroid comparison group and no association was detectable (TSH–HbA1c ρ = −0.13, *p* = 0.18; Supplementary Table S5).

*Sex-stratified analyses.* Among euthyroid and hypothyroid pattern participants, the TSH–HbA1c correlation was 0.66 (95% CI 0.55 to 0.74) in women (*n* = 161) and 0.74 (95% CI 0.66 to 0.80) in men (*n* = 102; difference *p* = 0.19); the TSH–FPG correlation was stronger in men (0.78 versus 0.64; *p* = 0.023). The ln TSH by sex interaction terms were not significant (HbA1c *p* = 0.11; FPG *p* = 0.14). Results in the whole cohort were similar (Supplementary Table S6 and Figure S2).

## 4. Discussion

This cross-sectional study of 331 adults from a two-source clinical laboratory sample in Jazan found that participants with a hypothyroid or hyperthyroid biochemical pattern had markedly higher HbA1c and FPG than euthyroid comparison participants, with strong correlations between TSH and both glycemic markers in the pooled data (ρ = 0.59 and 0.63). The central finding of the revised analysis, however, is that the comparison group had been selected for normal glucose values as well as normal thyroid function, so that the between-group difference was built into the design, and that the correlations did not exist within groups: among participants with an elevated TSH, neither TSH nor FT4 was related to HbA1c or FPG, overt and subclinical hypothyroid patterns had identical glycemic values, and thyroid function group alone explained nearly half (46%) of the variance in HbA1c, far more than the continuous thyroid parameters, age, sex and BMI together. The pooled association is therefore best understood as a difference between the two sampling sources rather than as a graded relationship between thyroid function and glycemia.

### 4.1. Interpretation Within a Cross-Sectional, Two-Source Design

The abnormal thyroid function group was drawn from hospital laboratory records, in which 88% of participants with HbA1c had a value of 5.7% or higher, whereas the euthyroid comparison group consisted of patients selected, by design, for thyroid function tests and glucose values within the reference intervals and was younger and leaner (median BMI 22.8 kg/m^2^); that 97% of its HbA1c values were normal is a consequence of the selection criteria, not a finding. Selecting a comparison group on the outcome guarantees a between-group difference in that outcome whatever the biological relationship between TSH and glucose, and combining two sources that differ in glycemic status, adiposity and referral pathway generates a large correlation across the pooled sample, a form of spectrum bias closely related to Berkson’s selection effect in hospital data [40,41]. The within-group analyses (Table 3, Figure 3) are the appropriate test of a graded association, and they were null. Sex, age and BMI did not account for the group difference, which persisted essentially unchanged in the group-indicator models.

Associations persisted whether TSH was modeled as a predictor of the glycemic markers or as the outcome (Supplementary Table S8). Because reversing outcome and predictor within the same cross-sectional dataset yields mathematically related estimates, this symmetry is a check on model specification rather than evidence of a bidirectional relationship, and it cannot distinguish between the possible explanations for the group difference: (1) thyroid dysfunction influencing glycemia through metabolic mechanisms; (2) dysglycemia influencing thyroid function through inflammatory or autoimmune pathways; (3) confounding by the sampling frame, by unmeasured clinical characteristics of hospital patients (including a prior diagnosis of diabetes and its treatment), or by adiposity, systemic inflammation or autoimmune disease; or (4) combinations thereof. All reported coefficients and correlations describe associations in this sample and carry no causal or directional implication. Cross-sectional data cannot distinguish among these scenarios; prospective longitudinal studies with repeated measures are essential to disentangle them.

### 4.2. Comparison with Prior Studies

Our pooled findings align in direction with prior cross-sectional studies reporting associations between TSH and HbA1c [20,22,23,24]. However, the magnitude of the pooled correlations (ρ ≈ 0.6) greatly exceeds the weak or absent correlations reported in unselected populations, in which higher HbA1c is generally not accompanied by higher TSH, and the absence of any within-group correlation here is consistent with that literature rather than with a strong biological coupling. Prospective data on thyroid function and glycemic trajectories in patients with established diabetes remain scarce [51], and the largest pooled analyses of thyroid function and incident diabetes have yielded inconsistent results [25,26,27], underscoring that the direction and even the existence of a population-level association remain unresolved. The features of the present cohort that inflated the pooled correlations, namely a hospital-derived abnormal-thyroid group with a very high prevalence of dysglycemia range values (32% with a diabetes range value on at least one marker) and a lean, normoglycemic comparison group, are shared to varying degrees by many laboratory-based studies, and may explain part of the heterogeneity between cross-sectional reports. These estimates should therefore not be extrapolated to unselected populations.

### 4.3. HbA1c Interpretation and Erythrocyte Kinetics

An alternative, non-glycemic explanation for part of the TSH–HbA1c association is the effect of thyroid function on erythrocyte turnover: hypothyroidism can prolong red cell lifespan and thereby raise HbA1c independently of ambient glycemia, whereas hyperthyroidism can exert the opposite effect [19,20,21]. The magnitude of this artifact is modest. In non-diabetic adults with overt hypothyroidism, restoring euthyroidism with levothyroxine lowered HbA1c from 5.8% to 5.6% over three months without any change in fasting or post-load glucose, and the proportion classified as dysglycemic by HbA1c fell from 66% to 45% [52]; Kim et al. similarly observed a fall in HbA1c with a concomitant rise in glycated albumin after replacement therapy [19], and comparable shifts of a few tenths of a percentage point in the opposite direction have been described in hyperthyroidism [20]. An erythrokinetic effect of 0.2 to 0.3 percentage points could therefore move borderline values across the 5.7% and 6.5% thresholds, but it cannot account for the 1.7-percentage-point difference between the hypothyroid pattern and euthyroid groups observed here, nor for the parallel difference in FPG (1.7 mmol/L), which is unaffected by red cell lifespan. The concordance between HbA1c and FPG thus argues against a purely analytical explanation but does not exclude a contribution from erythrokinetics, and, more importantly, neither marker was related to the degree of thyroid dysfunction within groups. The consistency of the two markers likewise cannot exclude confounding by the sampling frame, which would affect both markers equally. Corroborating HbA1c with plasma glucose-based measures is prudent when assessing glycemia in patients with overt thyroid dysfunction [19,52].

### 4.4. BMI as a Correlate

BMI correlated with HbA1c, FPG and TSH in the pooled data and was associated with both glycemic markers in the continuous whole-cohort models, but it was not associated with either marker within any thyroid function group, and its coefficient fell to zero once group membership was included. BMI in this cohort therefore behaved largely as a marker of the sampling source: the euthyroid comparison group was lean, whereas the abnormal thyroid function group had a median BMI in the overweight range. This is consistent with adiposity acting as a shared correlate of both thyroid function and glycemic control [5,31,33,36,37], and it underscores the importance of anthropometric adjustment in studies of thyroid–glucose relationships, while illustrating that adjustment cannot remove a difference that is built into the sampling design. The attenuation of the FT4 and FT3 associations in the adjusted models is likewise explained by the group structure, and mediation cannot be assessed in cross-sectional data.

### 4.5. Unmeasured Pharmacotherapy and Other Clinical Confounders

Medication data were not available, and several drug classes could have contributed to the biochemical patterns observed. Levothyroxine lowers TSH and, as described above, lowers HbA1c through erythrokinetic effects, so treated hypothyroid patients sampled during dose titration may show an elevated TSH with a partially corrected HbA1c; antithyroid drugs have the converse effect during treatment of hyperthyroidism. Metformin reduces TSH by roughly 1.1 to 1.6 mIU/L in patients with overt or subclinical hypothyroidism without affecting euthyroid individuals [38], which would attenuate rather than create a positive TSH–glycemia association, whereas glucocorticoids suppress TSH and raise glucose [39], which could generate hyperthyroid pattern participants with dysglycemia. Amiodarone, lithium and iodine-containing preparations alter thyroid function, and insulin and other glucose-lowering drugs alter glycemia in ways that depend on the underlying diagnosis. Because the abnormal thyroid function group was hospital-derived, a substantial proportion may have had established diabetes under treatment, so that the group difference in HbA1c and FPG may partly reflect the case-mix of the referring clinics rather than the thyroid function itself. Pregnancy, which alters TSH interpretation, was an exclusion criterion, but non-thyroidal illness at the time of sampling could not be excluded beyond the documented absence of acute illness. These considerations reinforce the descriptive interpretation of the findings.

### 4.6. Regional Endocrine Context

Two regional characteristics are relevant to the interpretation of thyroid function data from southwestern Saudi Arabia. First, iodine intake in Jazan is high: a school-based survey found a median urinary iodine concentration of 421 µg/L, with 74% of children above 300 µg/L, indicating more-than-adequate to excessive iodine intake that may predispose to iodine-induced hyperthyroidism and autoimmune thyroid disease [53], and thyroid abnormalities are frequent among adults tested in the neighboring Asir region [6]. Second, metabolic syndrome, obesity and type 2 diabetes are highly prevalent in the Saudi adult population [7,54], so that dysglycemia range values are expected to be common among hospital-attending adults regardless of thyroid function. Both features favor a high background prevalence of abnormal thyroid function and of dysglycemia in the source population, and they make the coexistence of the two conditions in hospital samples likely even in the absence of a graded biochemical relationship.

### 4.7. Autoimmune Confounding

Anti-thyroid peroxidase and anti-thyroglobulin antibodies were not measured, so autoimmune thyroid disease could not be distinguished from non-autoimmune causes of an abnormal thyroid function pattern, including iodine-related dysfunction, non-thyroidal illness and drug effects. Autoimmune thyroiditis and Graves’ disease share genetic and immunological risk factors with type 1 diabetes and are more common in people with type 2 diabetes than in the general population [30,32,33], so an autoimmune pathway linking the two conditions cannot be assessed in this dataset, and the etiological heterogeneity of the abnormal thyroid function group may itself have contributed to the absence of a dose–response relationship.

### 4.8. Study Limitations

*Cross-sectional design.* This is the most fundamental limitation: the design precludes causal inference, and temporal relationships cannot be established. Reversed regression models do not overcome this limitation. Prospective longitudinal studies are needed to determine directionality.

*Two-source sampling, selection on the outcome and spectrum bias.* The abnormal thyroid function group was selected from hospital laboratories on the basis of an abnormal thyroid result, and the euthyroid comparison group was selected at a single hospital for normal thyroid function and normal glucose values; the comparison group therefore cannot serve as an unselected reference, and the between-group differences in HbA1c and FPG are uninformative about the association of interest. The two sources also differed in adiposity, and the euthyroid group showed an unusually narrow HbA1c distribution (5.1% [4.9–5.2]). Pooled correlations and group differences therefore overstate any biological association and cannot be generalized to unselected populations or to consecutive laboratory patients. The absence of within-group associations is the more informative result.

*Missing data.* HbA1c was missing in 11.2% and FPG in 18.7% of participants, and missingness was concentrated in the hyperthyroid pattern group (44.6% for HbA1c and 83.9% for FPG) and at two of the three hospitals. Estimates for the hyperthyroid pattern group, in particular its FPG values, rest on very few observations and should be regarded as unreliable; the multiple-imputation analysis showed that the pooled TSH–FPG correlation and the continuous ln TSH–FPG coefficient are sensitive to the assumption made about the missing values, whereas the HbA1c results and the group differences were robust. Imputation assumes missingness at random, conditional on the included variables, and cannot correct for missingness related to unmeasured clinical factors.

*Absence of thyroid antibody data.* Anti-thyroid peroxidase and anti-thyroglobulin antibodies were not measured, precluding distinction between autoimmune and non-autoimmune thyroid dysfunction (Section 4.7).

*Unmeasured metabolic variables.* Insulin-resistance indices, inflammatory markers, and autoimmune markers were not available, limiting mechanistic interpretation. The modest R^2^ of the continuous models (0.18 for HbA1c and 0.38 for FPG) indicates that thyroid parameters, age, sex and BMI together account for only a small fraction of the variability of the glycemic markers, and that many unmeasured factors contribute; the statistically significant ln TSH coefficients correspond to small absolute effects (0.12 percentage points of HbA1c per doubling of TSH), comparable to the effect of about 2 kg/m^2^ of BMI, and should not be over-interpreted clinically.

*Absence of medication and diagnosis data.* Information on thyroid-modifying therapy (e.g., levothyroxine or antithyroid drugs), glucose-lowering therapy, and other medications known to influence thyroid function or glycemia (e.g., glucocorticoids, amiodarone, lithium, or oral contraceptives) was not available in the laboratory records, nor were prior clinical diagnoses of diabetes, thyroid disease or pituitary disease. Treated and untreated thyroid dysfunction therefore could not be distinguished, participants with known diabetes could not be identified or excluded, thyrotropin-secreting pituitary adenomas and other rare causes of an inappropriately elevated TSH could not be ruled out, and medication use could not be entered as a covariate (Section 4.5). The glycemic range restriction analyses were used in place of a diagnosis-based restriction; they condition on the outcome distribution and therefore necessarily attenuate associations, and their results are indicative only.

*Single-point, TSH-based classification.* Thyroid function was defined by a single TSH measurement, so transient non-thyroidal or assay-related variation could not be excluded, and the sub-classification into overt and subclinical patterns relied on FT4 alone. The 4.0 mIU/L threshold is lower than the manufacturer’s upper reference limit (5.60 mIU/L), but because only four participants had a TSH between 4.0 and 5.6 mIU/L the choice of threshold had no material effect on group composition, prevalence estimates or group comparisons (Section 3.11). A number of TSH and FT3 values lay at the limits of the analytical measuring range; these values do not affect rank-based statistics but were retained in the regression models, in which ln TSH was used to limit their influence. Because free thyroid hormone reference intervals are method-dependent, FT4 and FT3 values should be compared with other cohorts with caution.

*Sex-related heterogeneity.* Thyroid disease is more common in women, sex hormones modulate both thyroid hormone binding and insulin sensitivity, and the distribution of adiposity differs between sexes, so the association between thyroid function and glycemia could differ between women and men. In the exploratory sex-stratified analyses the TSH–HbA1c correlations were similar in women and men, the TSH–FPG correlation was somewhat stronger in men, and the ln TSH by sex interaction terms were not significant; however, the study was not powered for interaction tests, the hyperthyroid pattern group (56 participants, 30 women) was too small to stratify, and the stratified estimates share the sampling limitations of the pooled analysis. Sex-disaggregated analyses in larger, consecutively sampled cohorts are warranted.

### 4.9. Future Directions

The descriptive findings generate hypotheses rather than test them. Prospective studies with consecutive sampling from a single source, repeated measurements of thyroid function and glycemia, documentation of diagnoses and medication, and measurement of thyroid antibodies and insulin resistance are required to establish whether any temporal relationship exists between thyroid dysfunction and glycemic control in this population, and whether both conditions reflect shared metabolic, inflammatory or autoimmune pathways. The de-identified dataset and analysis code are available from the corresponding author on reasonable request to allow other groups to reproduce and extend these analyses.

## 5. Conclusions

In this cross-sectional study of adults with abnormal thyroid function tests and a euthyroid comparison group from Jazan, Saudi Arabia, participants with hypothyroid and hyperthyroid biochemical patterns had substantially higher HbA1c and FPG than euthyroid comparison participants, and TSH correlated strongly with both glycemic markers in the pooled data. These correlations were not present within thyroid function groups: among participants with an elevated TSH, neither TSH nor FT4 was related to glycemia, and overt and subclinical patterns had identical glycemic values.

The pooled associations therefore reflect the two-source sampling design, in which the comparison group was selected for normal glucose values and the clinical characteristics of hospital patients, rather than for a graded relationship between thyroid function and glucose metabolism, while the cross-sectional design precludes any inference about causality or direction. Clinicians should interpret concurrent abnormalities in thyroid and glycemic markers with caution, corroborate HbA1c with plasma glucose in patients with overt thyroid dysfunction, and not attribute glycemic dysfunction to thyroid disease, or vice versa, on the basis of cross-sectional associations. Prospective, consecutively sampled cohorts with within-person repeated measures are needed to establish whether a clinically meaningful relationship exists.

## Figures and Tables

**Figure 1:**
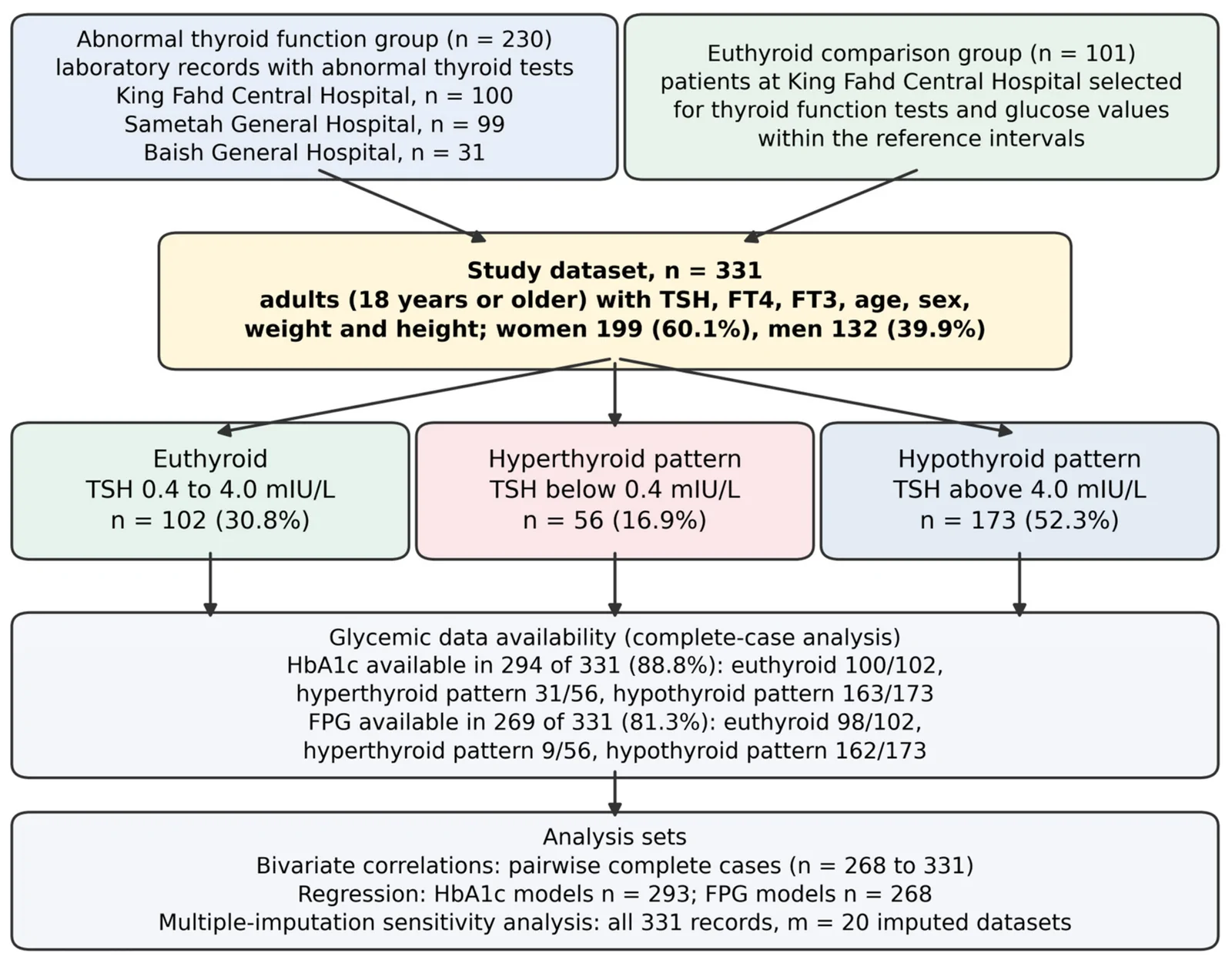
Data sources, participant classification and analysis sets. Records from two sources were combined into a dataset of 331 adults, classified by TSH into three thyroid function groups. Availability of HbA1c and FPG by group and the sample sizes of the complete-case regression models and of the multiple-imputation sensitivity analysis are shown.

**Figure 2:**
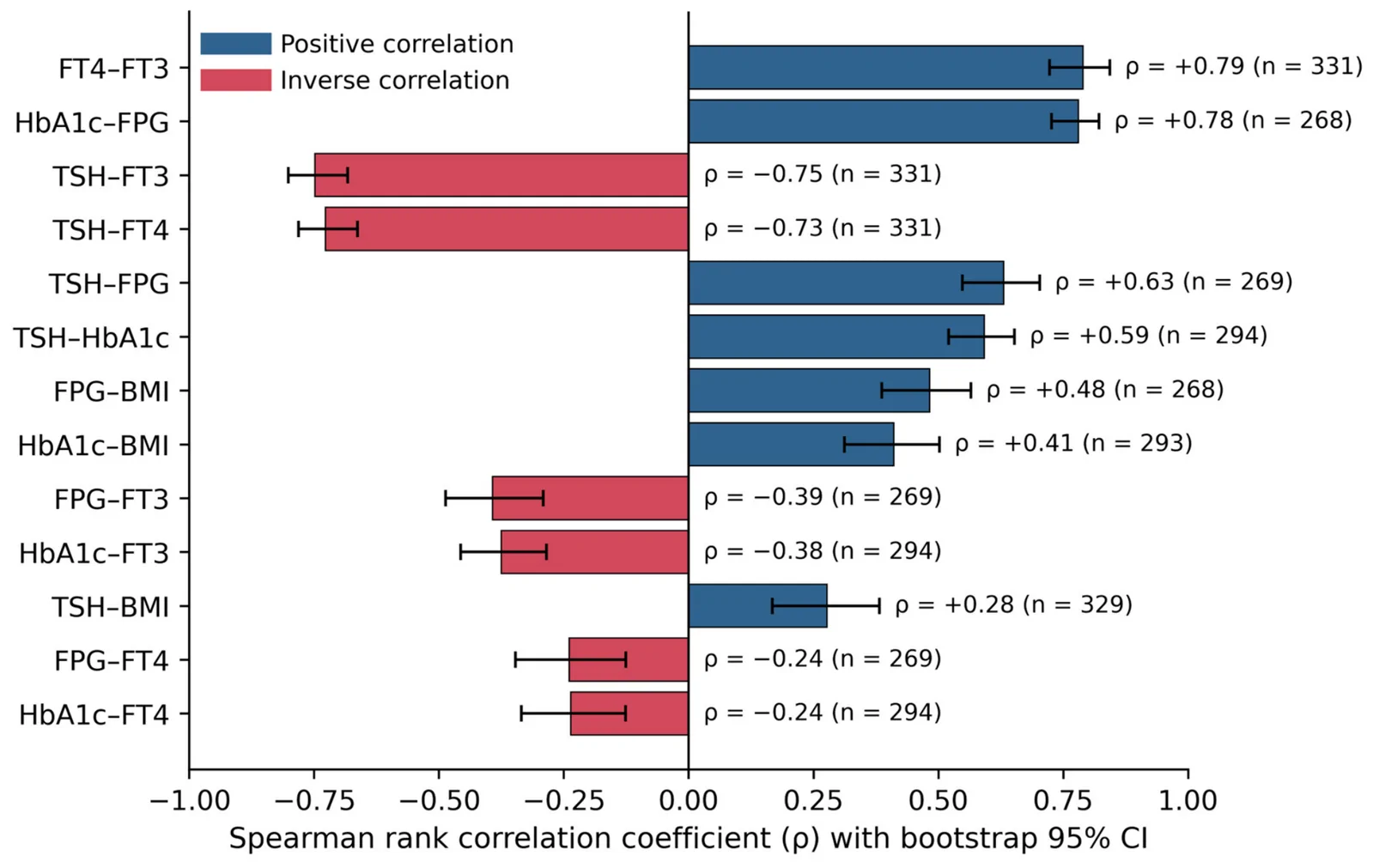
Bivariate Spearman rank correlations among thyroid and glycemic markers and BMI in the whole cohort, ordered by magnitude. Navy bars indicate positive correlations and crimson bars indicate inverse correlations; error bars are bootstrap 95% confidence intervals and the number of paired observations is given for each coefficient. All coefficients were statistically significant (*p* < 0.001; see Table 2).

**Figure 3:**
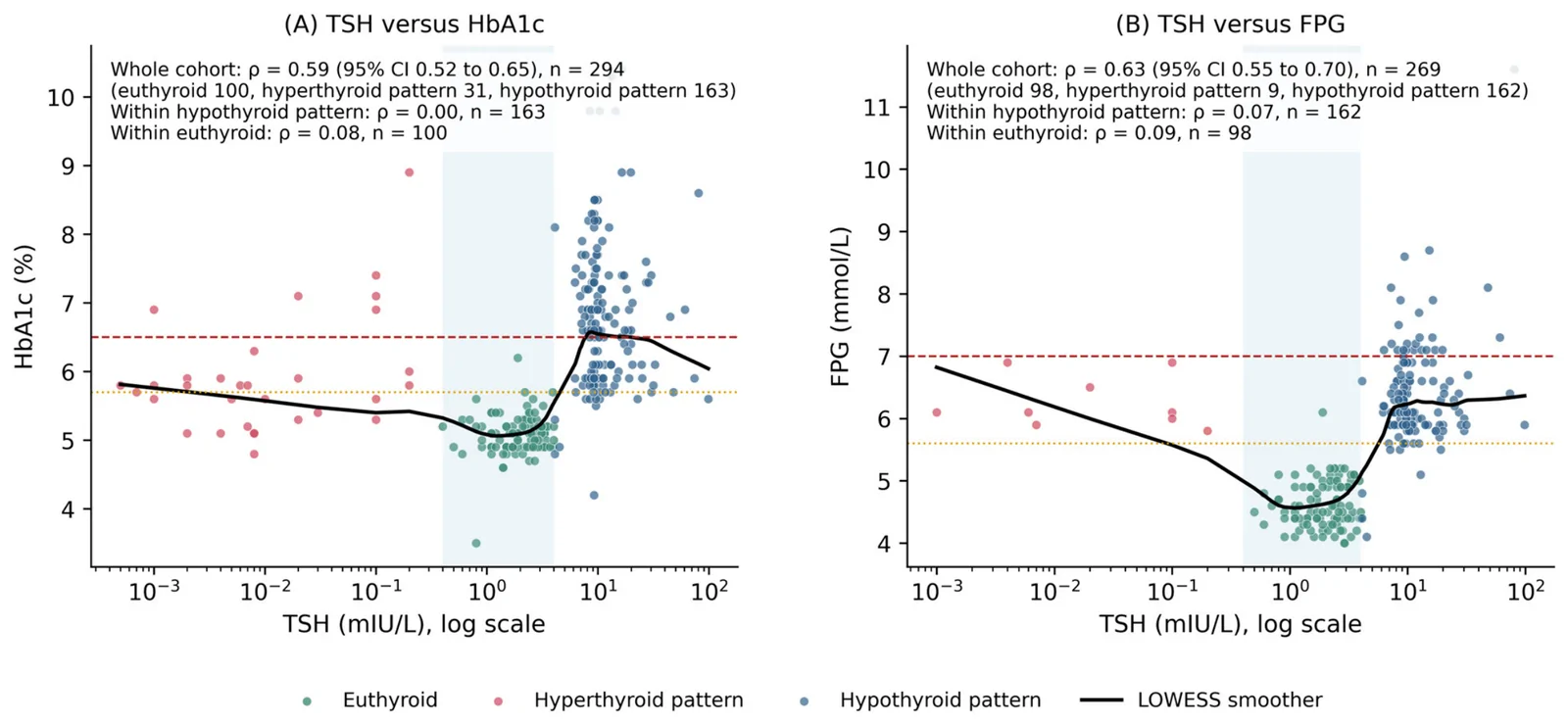
Scatter plots of TSH (logarithmic scale) against (**A**) HbA1c and (**B**) FPG, with one point per participant colored by thyroid function group and a locally weighted (LOWESS) smoother. The light blue vertical band indicates the euthyroid TSH range used for classification (0.4–4.0 mIU/L); dotted horizontal lines mark the prediabetes range (HbA1c 5.7%; FPG 5.6 mmol/L) and dashed lines the diabetes range (HbA1c 6.5%; FPG 7.0 mmol/L) thresholds. Whole-cohort and within-group Spearman coefficients are annotated. The plots show that the pooled correlation arises from the separation of the groups rather than from a graded relationship within any group.

**Figure 4:**
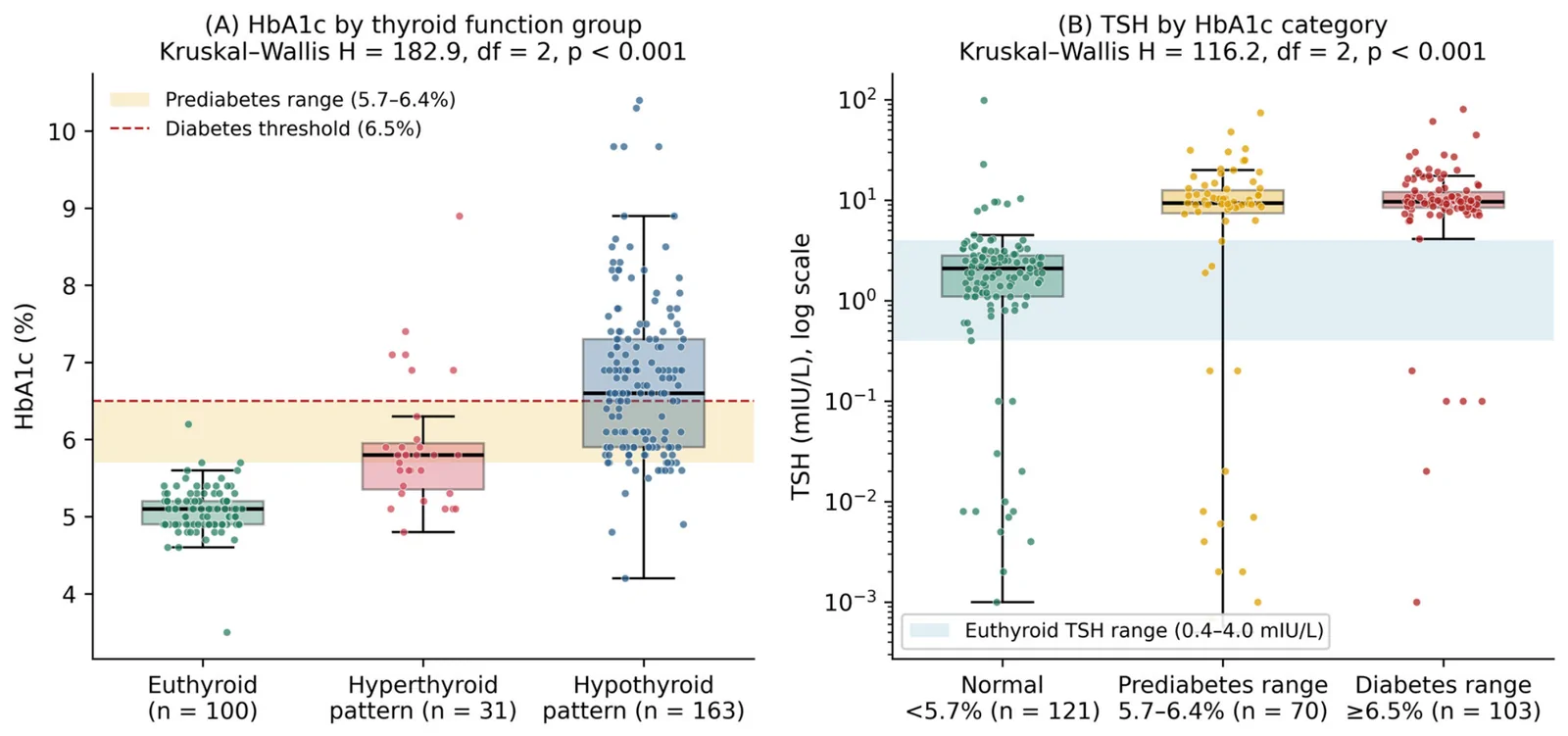
Glycemic and thyroid markers across laboratory-defined strata. (**A**) HbA1c by thyroid function group and (**B**) TSH (logarithmic scale) by HbA1c category. Boxes show the median and interquartile range, whiskers extend to the most extreme values within 1.5 times the interquartile range, and every participant is shown as a jittered point. Kruskal–Wallis test statistics are shown within each panel. In (**A**), the shaded band indicates the prediabetes HbA1c range (5.7–6.4%) and the dashed line the diabetes-range threshold (6.5%); in (**B**), the shaded band indicates the euthyroid TSH range used for classification (0.4–4.0 mIU/L).

**Figure 5:**
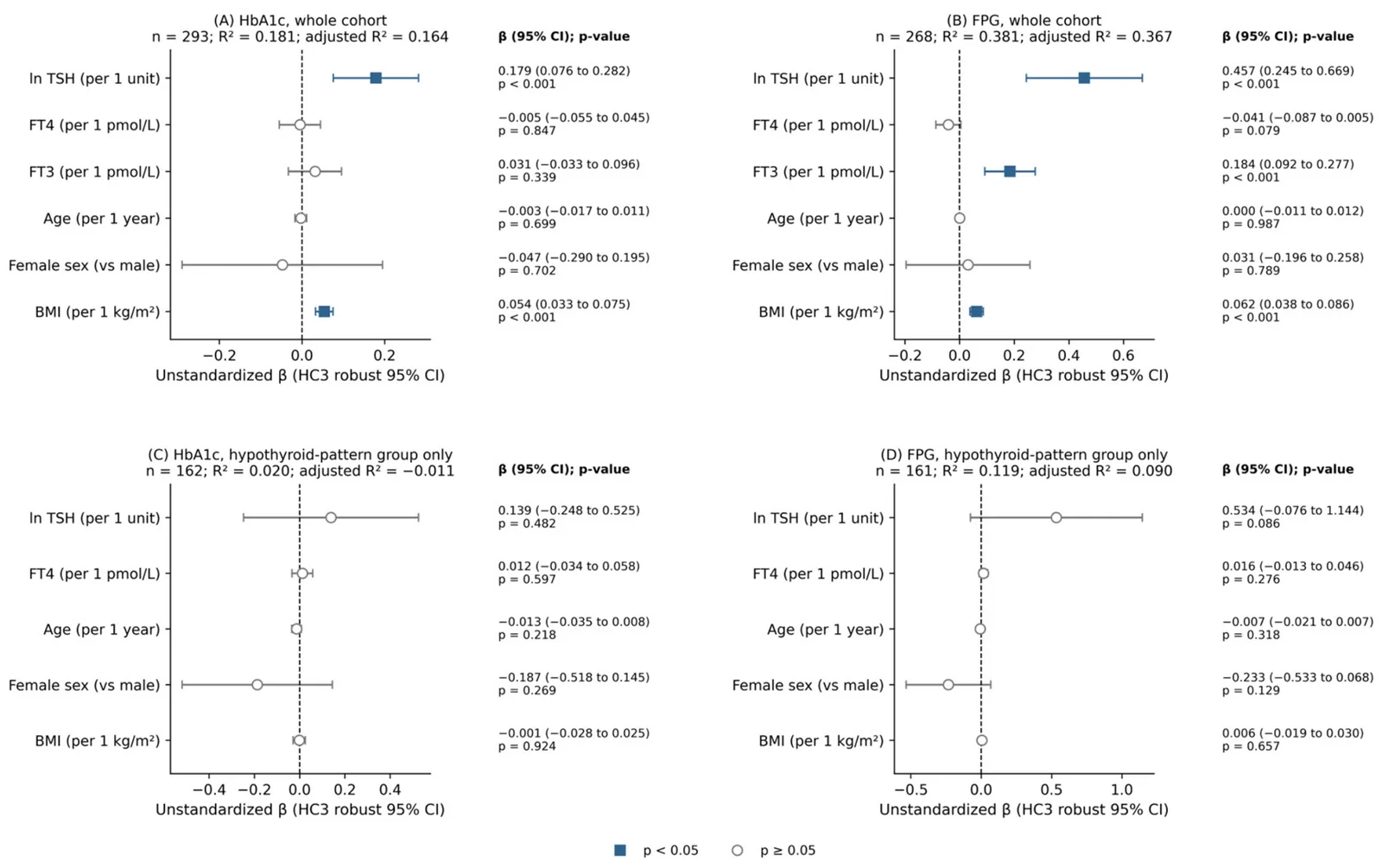
Adjusted linear regression coefficients (β) with HC3 robust 95% confidence intervals for the continuous models: (**A**) HbA1c and (**B**) FPG in the whole cohort; (**C**) HbA1c and (**D**) FPG within the hypothyroid pattern group. Filled squares denote predictors with *p* < 0.05 and open circles predictors with *p* ≥ 0.05; the dashed vertical line marks β = 0. Coefficients are shown per unit of each predictor (ln TSH per natural log unit) and correspond to Table 4, parts B and C.

**Table 1:** Characteristics of the study participants overall and by thyroid function group.

Variable	All Participants	Euthyroid	Hyperthyroid Pattern	Hypothyroid Pattern	*p*-Value
Participants, *n*	331	102	56	173	–
Women, *n* (%)	199 (60.1%)	54 (52.9%)	30 (53.6%)	115 (66.5%)	0.05 ^a^
Age (years)	37 [31–43]	36 [31–42]	37 [31–46]	37 [31–42]	0.789
TSH (mIU/L)	6.30 [1.45–9.90]	2.10 [1.40–2.70]	0.01 [0.00–0.02]	9.90 [8.90–13.20]	–
FT4 (pmol/L)	10.90 [6.30–12.90]	11.25 [10.22–12.17]	20.70 [17.88–29.40]	8.20 [4.40–11.10]	<0.001
FT3 (pmol/L)	4.80 [3.25–5.40]	4.90 [4.80–5.30]	10.15 [9.10–12.72]	3.70 [2.10–4.60]	<0.001
HbA1c (%)	5.90 [5.20–6.90] (*n* = 294)	5.10 [4.90–5.20] (*n* = 100)	5.80 [5.35–5.95] (*n* = 31)	6.60 [5.90–7.30] (*n* = 163)	<0.001
FPG (mmol/L)	5.90 [4.80–6.30] (*n* = 269)	4.50 [4.40–4.90] (*n* = 98)	6.10 [6.00–6.50] (*n* = 9)	6.10 [5.90–6.60] (*n* = 162)	<0.001
Weight (kg)	68.0 [60.0–75.0]	62.0 [56.2–70.0]	66.0 [60.0–78.0]	71.0 [64.0–80.0]	–
Height (cm)	163.0 [157.0–168.0] (*n* = 329)	166.0 [160.0–170.8]	160.0 [155.5–165.0]	161.0 [157.0–166.0]	–
BMI (kg/m^2^)	25.39 [22.50–29.41] (*n* = 329)	22.77 [20.71–24.09]	25.78 [22.13–30.33]	26.82 [24.29–31.23]	<0.001

Values are median [interquartile range] unless stated otherwise; *p*-values from the Kruskal–Wallis test across the three groups (^a^ chi-square test). Sample sizes for HbA1c, FPG, height and BMI are shown where data were incomplete. Means and standard deviations are given in Supplementary Table S1. TSH, thyroid-stimulating hormone; FT4, free thyroxine; FT3, free triiodothyronine; HbA1c, glycated hemoglobin; FPG, fasting plasma glucose; BMI, body mass index.

**Table 2:** Bivariate correlations between thyroid function parameters, glycemic markers and BMI in the whole cohort.

Variable Pair	*n*	Spearman ρ (95% CI)	*p*-Value	Pearson r	*p*-Value
TSH–HbA1c	294	+0.59 (+0.52 to +0.65)	<0.001	+0.33	<0.001
TSH–FPG	269	+0.63 (+0.55 to +0.70)	<0.001	+0.52	<0.001
TSH–FT4	331	−0.73 (−0.78 to −0.66)	<0.001	−0.44	<0.001
TSH–FT3	331	−0.75 (−0.80 to −0.68)	<0.001	−0.36	<0.001
FT4–FT3	331	+0.79 (+0.72 to +0.84)	<0.001	+0.93	<0.001
TSH–BMI	329	+0.28 (+0.17 to +0.38)	<0.001	+0.21	<0.001
HbA1c–FPG	268	+0.78 (+0.73 to +0.82)	<0.001	+0.69	<0.001
HbA1c–FT4	294	−0.24 (−0.33 to −0.13)	<0.001	−0.22	<0.001
HbA1c–FT3	294	−0.38 (−0.46 to −0.28)	<0.001	−0.20	<0.001
HbA1c–BMI	293	+0.41 (+0.31 to +0.50)	<0.001	+0.31	<0.001
FPG–FT4	269	−0.24 (−0.35 to −0.13)	<0.001	−0.22	<0.001
FPG–FT3	269	−0.39 (−0.49 to −0.29)	<0.001	−0.15	0.014
FPG–BMI	268	+0.48 (+0.39 to +0.57)	<0.001	+0.42	<0.001

ρ, Spearman rank correlation coefficient with bootstrap 95% confidence interval (2000 resamples); r, Pearson product–moment correlation coefficient; Kendall’s τ is given in Supplementary Table S7; abbreviations as in Table 1. Each variable pair is listed once.

**Table 3:** Spearman correlations within thyroid function groups and within the abnormal thyroid function group.

Variable Pair	Euthyroid	Hyperthyroid Pattern	Hypothyroid Pattern	Abnormal Thyroid Function Group ^a^
TSH–HbA1c	+0.08 (−0.12 to +0.29); *n* = 100	+0.24 (−0.13 to +0.55); *n* = 31	+0.00 (−0.16 to +0.15); *n* = 163	+0.24 (+0.09 to +0.37); *n* = 193 *
TSH–FPG	+0.09 (−0.11 to +0.29); *n* = 98	−0.35 (−0.87 to +0.47); *n* = 9	+0.07 (−0.09 to +0.24); *n* = 162	+0.02 (−0.12 to +0.18); *n* = 168
FT4–HbA1c	+0.08 (−0.12 to +0.28); *n* = 100	−0.41 (−0.64 to −0.07); *n* = 31 *	+0.08 (−0.07 to +0.23); *n* = 163	−0.21 (−0.36 to −0.06); *n* = 193 *
FT3–HbA1c	+0.40 (+0.20 to +0.58); *n* = 100 *	−0.32 (−0.59 to +0.03); *n* = 31	+0.06 (−0.10 to +0.22); *n* = 163	−0.20 (−0.35 to −0.04); *n* = 193 *
BMI–HbA1c	+0.09 (−0.12 to +0.28); *n* = 100	+0.15 (−0.24 to +0.49); *n* = 31	−0.02 (−0.18 to +0.14); *n* = 162	+0.01 (−0.13 to +0.16); *n* = 192
FT4–FPG	+0.02 (−0.17 to +0.22); *n* = 98	−0.13 (−0.81 to +0.75); *n* = 9	+0.02 (−0.13 to +0.16); *n* = 162	+0.01 (−0.12 to +0.16); *n* = 168
BMI–FPG	+0.05 (−0.14 to +0.24); *n* = 98	+0.47 (−0.49 to +0.94); *n* = 9	+0.10 (−0.07 to +0.25); *n* = 161	+0.08 (−0.09 to +0.23); *n* = 167
TSH–FT4	−0.18 (−0.36 to +0.00); *n* = 102	−0.53 (−0.72 to −0.27); *n* = 56 *	−0.44 (−0.54 to −0.31); *n* = 173 *	−0.76 (−0.82 to −0.69); *n* = 230 *

Values are Spearman ρ (bootstrap 95% CI); *n*, number of paired observations; * *p* < 0.05. ^a^ Hyperthyroid pattern and hypothyroid pattern participants combined (*n* = 230), i.e., excluding the euthyroid comparison group. Abbreviations as in Table 1.

**Table 4:** Multivariable linear regression models for HbA1c and FPG: (A) thyroid function group indicators, (B) continuous thyroid parameters in the whole cohort, and (C) continuous thyroid parameters within the hypothyroid pattern group.

Predictor	β	SE (HC3)	95% CI	*p*-Value	Standardized β
**(A) HbA1c~thyroid function group** (*n* = 293; R^2^ = 0.467; adjusted R^2^ = 0.458)					
Hyperthyroid pattern (vs. euthyroid)	0.838	0.168	0.509 to 1.168	<0.001	–
Hypothyroid pattern (vs. euthyroid)	1.682	0.103	1.480 to 1.885	<0.001	–
Age (years)	−0.004	0.006	−0.016 to 0.007	0.455	−0.03
Female sex (vs. male)	−0.129	0.096	−0.317 to 0.058	0.176	−0.06
BMI (kg/m^2^)	0.003	0.010	−0.017 to 0.022	0.800	0.01
**(A) FPG~thyroid function group** (*n* = 268; R^2^ = 0.623; adjusted R^2^ = 0.616)					
Hyperthyroid pattern (vs. euthyroid)	1.577	0.157	1.270 to 1.885	<0.001	–
Hypothyroid pattern (vs. euthyroid)	1.665	0.104	1.461 to 1.869	<0.001	–
Age (years)	−0.003	0.004	−0.011 to 0.006	0.521	−0.02
Female sex (vs. male)	−0.097	0.090	−0.274 to 0.080	0.283	−0.05
BMI (kg/m^2^)	0.011	0.010	−0.007 to 0.030	0.239	0.06
**(B) HbA1c~continuous thyroid parameters, whole cohort** (*n* = 293; R^2^ = 0.181; adjusted R^2^ = 0.164)					
ln TSH	0.179	0.053	0.076 to 0.282	<0.001	0.35
FT4 (pmol/L)	−0.005	0.025	−0.055 to 0.045	0.847	−0.03
FT3 (pmol/L)	0.031	0.033	−0.033 to 0.096	0.339	0.11
Age (years)	−0.003	0.007	−0.017 to 0.011	0.699	−0.02
Female sex (vs. male)	−0.047	0.124	−0.290 to 0.195	0.702	−0.02
BMI (kg/m^2^)	0.054	0.011	0.033 to 0.075	<0.001	0.27
**(B) FPG~continuous thyroid parameters, whole cohort** (*n* = 268; R^2^ = 0.381; adjusted R^2^ = 0.367)					
ln TSH	0.457	0.108	0.245 to 0.669	<0.001	0.63
FT4 (pmol/L)	−0.041	0.023	−0.087 to 0.005	0.079	−0.19
FT3 (pmol/L)	0.184	0.047	0.092 to 0.277	<0.001	0.53
Age (years)	0.000	0.006	−0.011 to 0.012	0.987	0.00
Female sex (vs. male)	0.031	0.116	−0.196 to 0.258	0.789	0.01
BMI (kg/m^2^)	0.062	0.012	0.038 to 0.086	<0.001	0.34
**(C) HbA1c, hypothyroid pattern group only** (*n* = 162; R^2^ = 0.020; adjusted R^2^ = −0.011)					
ln TSH	0.139	0.197	−0.248 to 0.525	0.482	0.07
FT4 (pmol/L)	0.012	0.023	−0.034 to 0.058	0.597	0.05
Age (years)	−0.013	0.011	−0.035 to 0.008	0.218	−0.11
Female sex (vs. male)	−0.187	0.169	−0.518 to 0.145	0.269	−0.09
BMI (kg/m^2^)	−0.001	0.014	−0.028 to 0.025	0.924	−0.01
**(C) FPG, hypothyroid pattern group only** (*n* = 161; R^2^ = 0.119; adjusted R^2^ = 0.090)					
ln TSH	0.534	0.311	−0.076 to 1.144	0.086	0.35
FT4 (pmol/L)	0.016	0.015	−0.013 to 0.046	0.276	0.08
Age (years)	−0.007	0.007	−0.021 to 0.007	0.318	−0.08
Female sex (vs. male)	−0.233	0.153	−0.533 to 0.068	0.129	−0.14
BMI (kg/m^2^)	0.006	0.012	−0.019 to 0.030	0.657	0.04

β, unstandardized regression coefficient (change in HbA1c in percentage points, or in FPG in mmol/L, per unit of the predictor); SE (HC3), heteroscedasticity-consistent standard error; CI, confidence interval; standardized β, change in the outcome in standard deviation units per standard deviation of the predictor (not defined for group indicators); ln TSH, natural logarithm of TSH (a coefficient multiplied by 0.693 gives the change per doubling of TSH). Model-based standard errors, bootstrap intervals, variance inflation factors and the symmetry check model are given in Supplementary Table S8. Other abbreviations as in Table 1.

## Data Availability

The de-identified participant-level dataset and the complete analysis code that generated every reported value are available upon reasonable request from the corresponding author, subject to institutional data-governance policies.

## Supplementary Materials

The following supporting information can be downloaded at https://www.mdpi.com/article/10.3390/medicina62101853/s1, Supplementary Table S1: Descriptive statistics (mean ± SD and median [IQR]) overall, by thyroid function group and by biochemical sub-pattern; Supplementary Table S2: Missing glycemic data by thyroid function group and site, logistic regression of HbA1c missingness, and comparison of hyperthyroid-pattern participants with and without HbA1c; Supplementary Table S3: Sensitivity of classification, prevalence estimates and group comparisons to the TSH thresholds; Supplementary Table S4: Multiple-imputation sensitivity analysis (chained equations with predictive mean matching, m = 20): pooled estimates compared with complete-case estimates; Supplementary Table S5: Sensitivity analyses restricted by laboratory-defined glycemic range (documented clinical diagnoses were not available); Supplementary Table S6: Sex-stratified Spearman correlations and ln TSH by sex interaction terms; Supplementary Table S7: Alternative correlation coefficients for all variable pairs in the whole cohort; Supplementary Table S8: Regression diagnostics and alternative specifications; Supplementary Figure S1: Regression residual diagnostics for the untransformed-TSH specification of the three original models (residuals versus fitted values, top; normal Q–Q plots of standardized residuals, bottom); Supplementary Figure S2: Sex-stratified scatter plots of TSH (logarithmic scale) against HbA1c in women and men, restricted to euthyroid and hypothyroid-pattern participants; each point is one participant.

## Abbreviations

The following abbreviations are used in this manuscript:

TSH	Thyroid-stimulating hormone (thyrotropin)
FT4	Free thyroxine
FT3	Free triiodothyronine
HbA1c	Glycated hemoglobin
FPG	Fasting plasma glucose
BMI	Body mass index
SD	Standard deviation
IQR	Interquartile range
SE	Standard error
CI	Confidence interval
HC3	Heteroscedasticity-consistent standard error, type 3
VIF	Variance inflation factor
LOWESS	Locally weighted scatterplot smoothing
CLIA	Chemiluminescent immunoassay
NGSP	National Glycohemoglobin Standardization Program
IFCC	International Federation of Clinical Chemistry and Laboratory Medicine
EQAS	External quality assurance scheme
CAP	College of American Pathologists
IRB	Institutional Review Board
STROBE	Strengthening the Reporting of Observational Studies in Epidemiology
