# Supplementary material for: Thyroid Function and Glycemic Profile in Adults with Abnormal Thyroid Function Tests and a Euthyroid Comparison Group: A Cross-Sectional Study from Jazan, Saudi Arabia
Source: Medicina (Kaunas). 2026 Sep 24;62(10):1853. doi: 10.3390/medicina62101853 (PMC13706782; doi:10.3390/medicina62101853)
Supplement: Supplementary file 1 [file medicina-62-01853-s001.zip › medicina-4549274-supplementary.pdf]

Supplementary Materials

Thyroid Function and Glycemic Profile in Adults with Abnormal Thyroid Function Tests and a Euthyroid Comparison Group: A Cross-Sectional Study from Jazan, Saudi Arabia

Mjery Y. et al. Manuscript medicina-4549274, revision 1. Contents: Tables S1–S8; Figures S1–S2. All values were generated by the analysis script from the verified study dataset (both available from the corresponding author on reasonable request).

Table S1. Descriptive statistics (mean ± SD and median [IQR]) overall, by thyroid function group and by biochemical sub-pattern.

Variable	All participants	Euthyroid	Hyperthyroid pattern	Hypothyroid pattern
Age	37.52 ± 9.26 / 37.00 [31.00–43.00] (n = 331)	36.93 ± 8.14 / 36.00 [31.00–42.00] (n = 102)	38.98 ± 12.22 / 37.00 [31.00–46.25] (n = 56)	37.39 ± 8.77 / 37.00 [31.00–42.00] (n = 173)
TSH	7.90 ± 11.02 / 6.30 [1.45–9.90] (n = 331)	2.08 ± 0.89 / 2.10 [1.40–2.70] (n = 102)	0.04 ± 0.06 / 0.01 [0.00–0.02] (n = 56)	13.88 ± 12.50 / 9.90 [8.90–13.20] (n = 173)
FT4	11.74 ± 7.88 / 10.90 [6.30–12.90] (n = 331)	11.30 ± 1.66 / 11.25 [10.22–12.17] (n = 102)	24.65 ± 10.27 / 20.70 [17.88–29.40] (n = 56)	7.83 ± 3.76 / 8.20 [4.40–11.10] (n = 173)
FT3	5.69 ± 5.13 / 4.80 [3.25–5.40] (n = 331)	5.17 ± 1.03 / 4.90 [4.80–5.30] (n = 102)	13.67 ± 8.24 / 10.15 [9.10–12.72] (n = 56)	3.41 ± 1.45 / 3.70 [2.10–4.60] (n = 173)
HbA1c	6.10 ± 1.14 / 5.90 [5.20–6.90] (n = 294)	5.08 ± 0.30 / 5.10 [4.90–5.20] (n = 100)	5.92 ± 0.86 / 5.80 [5.35–5.95] (n = 31)	6.76 ± 1.04 / 6.60 [5.90–7.30] (n = 163)
FPG	5.71 ± 1.05 / 5.90 [4.80–6.30] (n = 269)	4.63 ± 0.37 / 4.50 [4.40–4.90] (n = 98)	6.26 ± 0.41 / 6.10 [6.00–6.50] (n = 9)	6.34 ± 0.78 / 6.10 [5.90–6.60] (n = 162)
Weight	68.51 ± 13.92 / 68.00 [60.00–75.00] (n = 331)	62.00 ± 9.28 / 62.00 [56.25–70.00] (n = 102)	68.39 ± 14.25 / 66.00 [60.00–78.00] (n = 56)	72.39 ± 14.70 / 71.00 [64.00–80.00] (n = 173)
Height	162.35 ± 8.27 / 163.00 [157.00–168.00] (n = 329)	165.32 ± 8.71 / 166.00 [160.00–170.75] (n = 102)	160.58 ± 7.19 / 160.00 [155.50–165.00] (n = 55)	161.15 ± 7.90 / 161.00 [157.00–166.00] (n = 172)
BMI	26.12 ± 5.63 / 25.39 [22.50–29.41] (n = 329)	22.74 ± 3.45 / 22.77 [20.71–24.09] (n = 102)	26.68 ± 6.20 / 25.78 [22.13–30.33] (n = 55)	27.95 ± 5.59 / 26.82 [24.29–31.23] (n = 172)

Sub-pattern	n	TSH (mIU/L)	FT4 (pmol/L)	FT3 (pmol/L)	HbA1c (%)	HbA1c ≥6.5%, %	FPG (mmol/L)	BMI (kg/m²)
Euthyroid	102	2.10 [1.40–2.70]	11.25 [10.22–12.17]	4.90 [4.80–5.30]	5.1 [4.9–5.2] (n = 100)	0.0	4.5 [4.4–4.9] (n = 98)	22.8 [20.7–24.1]
Overt hyperthyroid pattern (high FT4 and/or FT3)	54	0.01 [0.00–0.02]	20.75 [18.30–30.00]	10.30 [9.10–12.97]	5.8 [5.3–5.9] (n = 29)	17.2	6.1 [6.0–6.2] (n = 8)	25.4 [22.0–30.3]
Overt hypothyroid pattern (low FT4)	86	12.55 [9.30–18.48]	4.40 [3.23–5.20]	2.10 [1.65–2.68]	6.6 [5.9–7.3] (n = 78)	55.1	6.1 [5.9–6.6] (n = 77)	27.5 [25.7–31.9]
Subclinical hyperthyroid pattern (normal FT4 and FT3)	2	0.05 [0.03–0.08]	12.35 [12.22–12.47]	4.70 [4.30–5.10]	6.8 [6.6–7.1] (n = 2)	50.0	6.9 [6.9–6.9] (n = 1)	31.2 [28.7–33.6]
Subclinical hypothyroid pattern (normal FT4)	87	9.30 [8.40–10.25]	11.10 [10.10–12.45]	4.60 [4.30–5.10]	6.6 [6.0–7.3] (n = 85)	63.5	6.1 [5.9–6.6] (n = 85)	26.2 [23.4–30.6]

Sub-patterns defined with the manufacturer's reference intervals (FT4 7.86–14.41 pmol/L; FT3 3.80–6.00 pmol/L). Overt versus subclinical hypothyroid pattern: HbA1c Mann–Whitney p = 0.73; FPG p = 0.80. Median [IQR] shown for sub-patterns.

Table S2. Missing glycemic data by thyroid function group and site, logistic regression of HbA1c missingness, and comparison of hyperthyroid-pattern participants with and without HbA1c.

Group / site	HbA1c missing	FPG missing
Euthyroid	2/102 (2.0%)	4/102 (3.9%)
Hyperthyroid pattern	25/56 (44.6%)	47/56 (83.9%)
Hypothyroid pattern	10/173 (5.8%)	11/173 (6.4%)
Site (abnormal thyroid function group): King Fahd Central Hospital	27/100 (27.0%)	38/100 (38.0%)
Site (abnormal group): Sametah General Hospital	10/99 (10.1%)	24/99 (24.2%)
Site (abnormal group): Baish General Hospital	0/31 (0%)	0/31 (0%)
Euthyroid comparison group (King Fahd Central Hospital; site field blank for 100 of 101)	0/101 (0%)	0/101 (0%)
Total	37/331 (11.2%)	62/331 (18.7%)

Predictor of missing HbA1c (logistic regression)	Odds ratio	p-value
Hyperthyroid pattern (vs euthyroid)	48.01	< 0.001
Hypothyroid pattern (vs euthyroid)	3.72	0.102
Age	0.99	0.485
Female	1.21	0.661
BMI	0.96	0.243

Hyperthyroid-pattern group	With HbA1c (n = 31), median	Without HbA1c (n = 25), median	Mann–Whitney p
Age	37.00	36.00	0.514
BMI	26.30	25.61	0.678
TSH	0.01	0.01	0.476
FT4	19.60	21.30	0.179
FT3	9.60	11.10	0.102
Women, %	51.6	56.0	–

Table S3. Sensitivity of classification, prevalence estimates and group comparisons to the TSH thresholds.

TSH limits (mIU/L)	Euthyroid n (%)	Hyperthyroid n (%)	Hypothyroid n (%)	HbA1c median: eu	hyper	hypo	HbA1c ≥6.5% (%): eu	hyper	hypo	KW H (HbA1c)	KW H (FPG)	χ^2
Prespecified (0.4-4.0)	102 (30.8%)	56 (16.9%)	173 (52.3%)	5.10	5.80	6.60	0.0	19.4	59.5	182.9	174.6	215.6
Alternative (0.4-4.5)	106 (32.0%)	56 (16.9%)	169 (51.1%)	5.10	5.80	6.60	1.0	19.4	60.4	187.8	180.8	222.4
Manufacturer (0.34-5.60)	106 (32.0%)	56 (16.9%)	169 (51.1%)	5.10	5.80	6.60	1.0	19.4	60.4	187.8	180.8	222.4

Participants with TSH between 4.0 and 5.6 mIU/L: n = 4 (all from the euthyroid comparison group; TSH 4.1, 4.1, 4.1 and 4.5 mIU/L; median HbA1c 5.1%, median FPG 4.6 mmol/L, median FT4 10.4 pmol/L). No participant had a TSH between 4.5 and 6.2 mIU/L or between 0.34 and 0.40 mIU/L. All p-values for the Kruskal–Wallis and chi-square tests were < 0.001 under every threshold.

Table S4. Multiple-imputation sensitivity analysis (chained equations with predictive mean matching, m = 20): pooled estimates compared with complete-case estimates.

Pair	n (complete case)	Complete-case ρ (95% CI)	Pooled ρ after imputation (95% CI)	p (pooled)
TSH - HbA1c	294	0.592 (0.521 to 0.653)	0.541 (0.450 to 0.621)	< 0.001
TSH - FPG	269	0.631 (0.548 to 0.703)	0.370 (0.239 to 0.488)	< 0.001
HbA1c - FPG	268	0.780 (0.726 to 0.822)	0.688 (0.611 to 0.752)	< 0.001

HbA1c - FT4	294	-0.236 (-0.335 to -0.126)	-0.248 (-0.353 to -0.137)	< 0.001
HbA1c - FT3	294	-0.375 (-0.456 to -0.284)	-0.357 (-0.456 to -0.251)	< 0.001
HbA1c - BMI	293	0.411 (0.312 to 0.502)	0.367 (0.265 to 0.460)	< 0.001

**Model**	**Term**	**Complete case: β (HC3 95% CI); p**	**Pooled after imputation: β (95% CI); p**	**Fraction of missing information**
A1 HbA1c ~ group	Hyperthyroid pattern	0.838 (0.509 to 1.168); p < 0.001	0.767 (0.435 to 1.098); p < 0.001	0.28
A1 HbA1c ~ group	Hypothyroid pattern	1.682 (1.480 to 1.885); p < 0.001	1.673 (1.442 to 1.904); p < 0.001	0.05
A1 HbA1c ~ group	Age	-0.004 (-0.016 to 0.007); p 0.455	-0.002 (-0.013 to 0.010); p 0.747	0.26
A1 HbA1c ~ group	Female	-0.129 (-0.317 to 0.058); p 0.176	-0.144 (-0.337 to 0.050); p 0.145	0.07
A1 HbA1c ~ group	BMI	0.003 (-0.017 to 0.022); p 0.800	0.002 (-0.017 to 0.020); p 0.835	0.09
A2 FPG ~ group	Hyperthyroid pattern	1.577 (1.270 to 1.885); p < 0.001	1.400 (1.002 to 1.799); p < 0.001	0.66
A2 FPG ~ group	Hypothyroid pattern	1.665 (1.461 to 1.869); p < 0.001	1.603 (1.417 to 1.789); p < 0.001	0.06
A2 FPG ~ group	Age	-0.003 (-0.011 to 0.006); p 0.521	-0.004 (-0.012 to 0.005); p 0.410	0.19
A2 FPG ~ group	Female	-0.097 (-0.274 to 0.080); p 0.283	-0.105 (-0.270 to 0.059); p 0.207	0.18
A2 FPG ~ group	BMI	0.011 (-0.007 to 0.030); p 0.239	0.013 (-0.002 to 0.028); p 0.087	0.14
B1 HbA1c ~ logTSH pooled	logTSH	0.179 (0.076 to 0.282); p < 0.001	0.148 (0.069 to 0.227); p < 0.001	0.15
B1 HbA1c ~ logTSH pooled	FT4	-0.005 (-0.055 to 0.045); p 0.847	-0.006 (-0.050 to 0.038); p 0.796	0.11
B1 HbA1c ~ logTSH pooled	FT3	0.031 (-0.033 to 0.096); p 0.339	0.029 (-0.036 to 0.094); p 0.378	0.20
B1 HbA1c ~ logTSH pooled	Age	-0.003 (-0.017 to 0.011); p 0.699	-0.000 (-0.013 to 0.013); p 0.987	0.17
B1 HbA1c ~ logTSH pooled	Female	-0.047 (-0.290 to 0.195); p 0.702	-0.051 (-0.286 to 0.184); p 0.669	0.05
B1 HbA1c ~ logTSH pooled	BMI	0.054 (0.033 to 0.075); p < 0.001	0.049 (0.028 to 0.069); p < 0.001	0.04
B2 FPG ~ logTSH pooled	logTSH	0.457 (0.245 to 0.669); p < 0.001	0.081 (-0.012 to 0.174); p 0.085	0.50
B2 FPG ~ logTSH pooled	FT4	-0.041 (-0.087 to 0.005); p 0.079	-0.018 (-0.066 to 0.029); p 0.451	0.37
B2 FPG ~ logTSH pooled	FT3	0.184 (0.092 to 0.277); p < 0.001	0.063 (-0.001 to 0.127); p 0.055	0.33
B2 FPG ~ logTSH pooled	Age	0.000 (-0.011 to 0.012); p 0.987	-0.001 (-0.013 to 0.011); p 0.856	0.11
B2 FPG ~ logTSH pooled	Female	0.031 (-0.196 to 0.258); p 0.789	-0.018 (-0.234 to 0.198); p 0.871	0.10
B2 FPG ~ logTSH pooled	BMI	0.062 (0.038 to 0.086); p < 0.001	0.063 (0.045 to 0.082); p < 0.001	0.05

Imputed group medians (mean of medians across imputations): HbA1c euthyroid 5.10%, hyperthyroid pattern 5.76%, hypothyroid pattern 6.61%; FPG 4.60, 6.10 and 6.10 mmol/L. Kruskal–Wallis H for HbA1c across imputed datasets ranged from 187.4 to 199.2 (complete case 182.9). Imputation model: HbA1c, FPG, ln TSH, FT4, FT3, age, sex, BMI, thyroid function group and site.

Table S5. Sensitivity analyses restricted by laboratory-defined glycemic range (documented clinical diagnoses were not available).

**Subset**	**n**	**Euthyroid/hyper/hypo with HbA1c**	**TSH–HbA1c ρ (95% CI)**	**TSH–FPG ρ (95% CI)**	**ln TSH β for HbA1c (HC3 95% CI)**	**ln TSH β for FPG (HC3 95% CI)**	**Kruskal–Wallis HbA1c**
Full cohort	331	100/31/163	0.59 (0.51 to 0.65); n = 294	0.63 (0.55 to 0.70); n = 269	0.179 (0.076 to 0.282); p <	0.457 (0.245 to 0.669); p <	H = 182.9; medians

					0.001; R^2 = 0.18	0.001; R^2 = 0.38	5.1/5.8/6.6
Excluding diabetes-range HbA1c (>=6.5%) or FPG (>=7.0 mmol/L)	224	100/25/62	0.44 (0.30 to 0.57); n = 187	0.61 (0.48 to 0.71); n = 166	0.035 (-0.027 to 0.097); p 0.269; R^2 = 0.23	0.391 (0.177 to 0.604); p < 0.001; R^2 = 0.51	H = 101.6; medians 5.1/5.6/5.9
Normoglycaemic range only (HbA1c <5.7% and FPG <5.6 mmol/L)	113	97/13/3	-0.13 (-0.32 to 0.06); n = 113	0.04 (-0.14 to 0.22); n = 98	-0.036 (-0.084 to 0.012); p 0.146; R^2 = 0.09	0.031 (-0.108 to 0.169); p 0.667; R^2 = 0.01	H = 8.7; medians 5.1/5.3/4.9

Restriction conditions on the outcome distribution and therefore attenuates associations by construction; the normoglycemic subset contains almost no participants with an abnormal thyroid pattern and cannot test the association.

Table S6. Sex-stratified Spearman correlations and ln TSH by sex interaction terms.

Participants	Pair	Women: ρ (95% CI)	Men: ρ (95% CI)	Difference (Fisher z)
Euthyroid + hypothyroid pattern	TSH - HbA1c	0.66 (0.55 to 0.74); n = 161	0.74 (0.66 to 0.80); n = 102	z = -1.32; p = 0.186
Euthyroid + hypothyroid pattern	TSH - FPG	0.64 (0.52 to 0.72); n = 159	0.78 (0.72 to 0.83); n = 101	z = -2.27; p = 0.023
Euthyroid + hypothyroid pattern	FT4 - HbA1c	-0.17 (-0.32 to -0.03); n = 161	-0.35 (-0.53 to -0.18); n = 102	z = 1.51; p = 0.132
Euthyroid + hypothyroid pattern	FT3 - HbA1c	-0.35 (-0.47 to -0.23); n = 161	-0.47 (-0.59 to -0.32); n = 102	z = 1.12; p = 0.261
Euthyroid + hypothyroid pattern	BMI - HbA1c	0.38 (0.23 to 0.51); n = 161	0.53 (0.37 to 0.66); n = 101	z = -1.48; p = 0.139
Euthyroid + hypothyroid pattern	TSH - BMI	0.40 (0.27 to 0.52); n = 169	0.50 (0.34 to 0.63); n = 105	z = -0.94; p = 0.345
All participants	TSH - HbA1c	0.60 (0.51 to 0.67); n = 177	0.57 (0.44 to 0.69); n = 117	z = 0.32; p = 0.751
All participants	TSH - FPG	0.55 (0.42 to 0.65); n = 166	0.72 (0.60 to 0.80); n = 103	z = -2.33; p = 0.020
All participants	FT4 - HbA1c	-0.19 (-0.30 to -0.06); n = 177	-0.27 (-0.44 to -0.09); n = 117	z = 0.77; p = 0.443
All participants	FT3 - HbA1c	-0.34 (-0.45 to -0.23); n = 177	-0.39 (-0.52 to -0.23); n = 117	z = 0.47; p = 0.640
All participants	BMI - HbA1c	0.33 (0.19 to 0.46); n = 177	0.51 (0.37 to 0.63); n = 116	z = -1.76; p = 0.079
All participants	TSH - BMI	0.20 (0.06 to 0.34); n = 198	0.38 (0.22 to 0.53); n = 131	z = -1.72; p = 0.085

Interaction terms (HC3): HbA1c model ln TSH × female β = 0.100 (95% CI -0.024 to 0.225), p = 0.11; FPG model β = -0.286 (95% CI -0.662 to 0.090), p = 0.14.

Table S7. Alternative correlation coefficients for all variable pairs in the whole cohort.

Pair	n	Spearman ρ	p	Pearson r	p	Kendall τ	p
TSH - HbA1c	294	+0.592	< 0.001	+0.331	< 0.001	+0.389	< 0.001
TSH - FPG	269	+0.631	< 0.001	+0.520	< 0.001	+0.437	< 0.001
TSH - FT4	331	-0.728	< 0.001	-0.438	< 0.001	-0.544	< 0.001
TSH - FT3	331	-0.748	< 0.001	-0.362	< 0.001	-0.566	< 0.001
FT4 - FT3	331	+0.790	< 0.001	+0.927	< 0.001	+0.621	< 0.001
TSH - BMI	329	+0.277	< 0.001	+0.207	< 0.001	+0.185	< 0.001
HbA1c - FPG	268	+0.780	< 0.001	+0.685	< 0.001	+0.589	< 0.001
HbA1c - FT4	294	-0.236	< 0.001	-0.219	< 0.001	-0.155	< 0.001
HbA1c - FT3	294	-0.375	< 0.001	-0.198	< 0.001	-0.229	< 0.001
HbA1c - BMI	293	+0.411	< 0.001	+0.308	< 0.001	+0.279	< 0.001
FPG - FT4	269	-0.239	< 0.001	-0.218	< 0.001	-0.164	< 0.001
FPG - FT3	269	-0.393	< 0.001	-0.150	0.014	-0.261	< 0.001

FPG - BMI	268	+0.483	< 0.001	+0.421	< 0.001	+0.333	< 0.001

Table S8. Regression diagnostics and alternative specifications.

(a) Primary ln TSH models: model-based versus HC3 standard errors, bootstrap intervals and variance inflation factors.

Model	Term	β	Model-based SE; p	HC3 SE; p	Bootstrap 95% CI	VIF
B1 HbA1c ~ logTSH pooled (n = 293)	logTSH	0.179	0.045; p < 0.001	0.053; p < 0.001	0.093 to 0.292	2.75
B1 HbA1c ~ logTSH pooled (n = 293)	FT4	-0.005	0.025; p 0.847	0.025; p 0.847	-0.050 to 0.048	7.51
B1 HbA1c ~ logTSH pooled (n = 293)	FT3	0.031	0.040; p 0.431	0.033; p 0.339	-0.036 to 0.094	6.86
B1 HbA1c ~ logTSH pooled (n = 293)	Age	-0.003	0.007; p 0.704	0.007; p 0.699	-0.017 to 0.012	1.01
B1 HbA1c ~ logTSH pooled (n = 293)	Female	-0.047	0.126; p 0.708	0.124; p 0.702	-0.300 to 0.188	1.03
B1 HbA1c ~ logTSH pooled (n = 293)	BMI	0.054	0.011; p < 0.001	0.011; p < 0.001	0.033 to 0.074	1.03
B2 FPG ~ logTSH pooled (n = 268)	logTSH	0.457	0.055; p < 0.001	0.108; p < 0.001	0.301 to 0.701	2.47
B2 FPG ~ logTSH pooled (n = 268)	FT4	-0.041	0.025; p 0.108	0.023; p 0.079	-0.084 to 0.006	5.82
B2 FPG ~ logTSH pooled (n = 268)	FT3	0.184	0.042; p < 0.001	0.047; p < 0.001	0.075 to 0.279	6.02
B2 FPG ~ logTSH pooled (n = 268)	Age	0.000	0.006; p 0.987	0.006; p 0.987	-0.011 to 0.012	1.02
B2 FPG ~ logTSH pooled (n = 268)	Female	0.031	0.106; p 0.770	0.116; p 0.789	-0.192 to 0.248	1.02
B2 FPG ~ logTSH pooled (n = 268)	BMI	0.062	0.009; p < 0.001	0.012; p < 0.001	0.036 to 0.083	1.04
C1 HbA1c within hypothyroid pattern (n = 162)	logTSH	0.139	0.179; p 0.441	0.197; p 0.482	-0.214 to 0.518	1.28
C1 HbA1c within hypothyroid pattern (n = 162)	FT4	0.012	0.025; p 0.616	0.023; p 0.597	-0.033 to 0.056	1.28
C1 HbA1c within hypothyroid pattern (n = 162)	Age	-0.013	0.010; p 0.184	0.011; p 0.218	-0.035 to 0.007	1.04
C1 HbA1c within hypothyroid pattern (n = 162)	Female	-0.187	0.175; p 0.288	0.169; p 0.269	-0.512 to 0.159	1.02
C1 HbA1c within hypothyroid pattern (n = 162)	BMI	-0.001	0.015; p 0.930	0.014; p 0.924	-0.026 to 0.025	1.02
C2 FPG within hypothyroid pattern (n = 161)	logTSH	0.534	0.131; p < 0.001	0.311; p 0.086	0.062 to 1.096	1.28
C2 FPG within hypothyroid pattern (n = 161)	FT4	0.016	0.018; p 0.359	0.015; p 0.276	-0.012 to 0.047	1.29
C2 FPG within hypothyroid pattern (n = 161)	Age	-0.007	0.007; p 0.320	0.007; p 0.318	-0.020 to 0.005	1.06
C2 FPG within hypothyroid pattern (n = 161)	Female	-0.233	0.126; p 0.066	0.153; p 0.129	-0.513 to 0.054	1.02
C2 FPG within hypothyroid pattern (n = 161)	BMI	0.006	0.011; p 0.603	0.012; p 0.657	-0.018 to 0.028	1.03

Model	n	R^2	Maximum Cook's D	Observations with Cook's D > 4/n	Shapiro–Wilk (residuals) p	Breusch–Pagan p
A1 HbA1c ~	293	0.467	0.090	15	< 0.001	< 0.001

group						
A2 FPG ~ group	268	0.623	0.210	10	< 0.001	0.220
B1 HbA1c ~ logTSH pooled	293	0.181	0.086	17	< 0.001	0.622
B2 FPG ~ logTSH pooled	268	0.381	0.265	13	< 0.001	0.873
C1 HbA1c within hypothyroid pattern	162	0.020	0.077	10	< 0.001	0.364
C2 FPG within hypothyroid pattern	161	0.119	0.930	8	< 0.001	0.005

(b) Re-estimation after excluding observations with Cook's distance > 4/n (HC3 standard errors).

Model	Term	n (all)	β (all)	n (excluding influential)	β (excluding)	95% CI (excluding)	p (excluding)
B1 HbA1c, whole cohort	logTSH	293	0.179	276	0.225	0.127 to 0.323	< 0.001
B1 HbA1c, whole cohort	BMI	293	0.054	276	0.058	0.040 to 0.077	< 0.001
B2 FPG, whole cohort	logTSH	268	0.457	255	0.719	0.618 to 0.821	< 0.001
B2 FPG, whole cohort	BMI	268	0.062	255	0.037	0.016 to 0.058	< 0.001
C1 HbA1c, hypothyroid pattern	logTSH	162	0.139	152	0.093	-0.225 to 0.412	0.565
C1 HbA1c, hypothyroid pattern	BMI	162	-0.001	152	-0.002	-0.024 to 0.020	0.835
C2 FPG, hypothyroid pattern	logTSH	161	0.534	153	0.228	0.012 to 0.443	0.039
C2 FPG, hypothyroid pattern	BMI	161	0.006	153	0.005	-0.011 to 0.021	0.526

(c) Models without FT3 (ln TSH, FT4, age, sex, BMI) and symmetry-check model with ln TSH as the outcome.

Model	Term	β	HC3 95% CI	p	Standardized β
B1x HbA1c ~ logTSH pooled, no FT3 (n = 293; R^2 = 0.179)	logTSH	0.173	0.071 to 0.275	< 0.001	0.34
B1x HbA1c ~ logTSH pooled, no FT3 (n = 293; R^2 = 0.179)	FT4	0.011	-0.020 to 0.042	0.473	0.06
B1x HbA1c ~ logTSH pooled, no FT3 (n = 293; R^2 = 0.179)	Age	-0.003	-0.017 to 0.011	0.697	-0.02
B1x HbA1c ~ logTSH pooled, no FT3 (n = 293; R^2 = 0.179)	Female	-0.038	-0.279 to 0.203	0.759	-0.02
B1x HbA1c ~ logTSH pooled, no FT3 (n = 293; R^2 = 0.179)	BMI	0.054	0.033 to 0.075	< 0.001	0.27
B2x FPG ~ logTSH pooled, no FT3 (n = 268; R^2 = 0.335)	logTSH	0.387	0.202 to 0.573	< 0.001	0.53
B2x FPG ~ logTSH pooled, no FT3 (n =	FT4	0.047	0.006 to 0.088	0.026	0.22

268; R^2 = 0.335)					
B2x FPG ~ logTSH pooled, no FT3 (n = 268; R^2 = 0.335)	Age	0.001	-0.012 to 0.013	0.922	0.00
B2x FPG ~ logTSH pooled, no FT3 (n = 268; R^2 = 0.335)	Female	0.064	-0.165 to 0.294	0.584	0.03
B2x FPG ~ logTSH pooled, no FT3 (n = 268; R^2 = 0.335)	BMI	0.064	0.040 to 0.088	< 0.001	0.35
D logTSH ~ glycaemia (symmetry check) (n = 267; R^2 = 0.210)	HbA1c	0.229	0.064 to 0.393	0.006	0.18
D logTSH ~ glycaemia (symmetry check) (n = 267; R^2 = 0.210)	FPG	0.434	0.291 to 0.578	< 0.001	0.31
D logTSH ~ glycaemia (symmetry check) (n = 267; R^2 = 0.210)	Age	-0.003	-0.028 to 0.022	0.817	-0.02
D logTSH ~ glycaemia (symmetry check) (n = 267; R^2 = 0.210)	Female	-0.020	-0.312 to 0.272	0.891	-0.01
D logTSH ~ glycaemia (symmetry check) (n = 267; R^2 = 0.210)	BMI	-0.001	-0.043 to 0.042	0.976	-0.00

(d) Original specification with untransformed TSH (as in the submitted version): model-based versus HC3 inference.

Model	Term	β	Model-based SE; p	HC3 SE; p	Bootstrap 95% CI	VIF
Model 1 (HbA1c) (n = 293; R^2 = 0.177; max Cook's D 0.64; Breusch–Pagan p 0.008)	TSH	0.024	0.006; p < 0.001	0.014; p 0.094	0.007 to 0.061	1.31
Model 1 (HbA1c) (n = 293; R^2 = 0.177; max Cook's D 0.64; Breusch–Pagan p 0.008)	FT4	-0.011	0.025; p 0.647	0.025; p 0.642	-0.054 to 0.044	7.26
Model 1 (HbA1c) (n = 293; R^2 = 0.177; max Cook's D 0.64; Breusch–Pagan p 0.008)	FT3	-0.012	0.040; p 0.766	0.035; p 0.739	-0.092 to 0.048	6.78
Model 1 (HbA1c) (n = 293; R^2 = 0.177; max Cook's D 0.64; Breusch–Pagan p 0.008)	Age	-0.005	0.007; p 0.511	0.008; p 0.548	-0.020 to 0.010	1.01
Model 1 (HbA1c) (n = 293; R^2 = 0.177; max Cook's D 0.64; Breusch–Pagan p 0.008)	Female	-0.008	0.125; p 0.946	0.120; p 0.944	-0.234 to 0.211	1.02
Model 1 (HbA1c) (n = 293; R^2 = 0.177; max Cook's D 0.64; Breusch–Pagan p 0.008)	BMI	0.050	0.011; p < 0.001	0.012; p < 0.001	0.028 to 0.071	1.07
Model 2 (FPG) (n = 268; R^2 = 0.367; max Cook's D 1.74; Breusch–Pagan p < 0.001)	TSH	0.042	0.005; p < 0.001	0.021; p 0.052	0.017 to 0.081	1.31
Model 2 (FPG) (n = 268; R^2 = 0.367; max Cook's D 1.74; Breusch–Pagan p < 0.001)	FT4	-0.023	0.026; p 0.377	0.026; p 0.378	-0.071 to 0.032	6.12

Model 2 (FPG) (n = 268; R^2 = 0.367; max Cook's D 1.74; Breusch–Pagan p < 0.001)	FT3	0.044	0.041; p 0.285	0.032; p 0.175	-0.044 to 0.104	5.64
Model 2 (FPG) (n = 268; R^2 = 0.367; max Cook's D 1.74; Breusch–Pagan p < 0.001)	Age	-0.005	0.006; p 0.444	0.007; p 0.522	-0.018 to 0.007	1.01
Model 2 (FPG) (n = 268; R^2 = 0.367; max Cook's D 1.74; Breusch–Pagan p < 0.001)	Female	0.049	0.107; p 0.647	0.112; p 0.661	-0.161 to 0.259	1.01
Model 2 (FPG) (n = 268; R^2 = 0.367; max Cook's D 1.74; Breusch–Pagan p < 0.001)	BMI	0.058	0.009; p < 0.001	0.011; p < 0.001	0.038 to 0.076	1.07
Model 3 (TSH) (n = 267; R^2 = 0.276; max Cook's D 0.81; Breusch–Pagan p 0.053)	HbA1c	-0.579	0.695; p 0.405	0.846; p 0.493	-2.165 to 0.876	1.89
Model 3 (TSH) (n = 267; R^2 = 0.276; max Cook's D 0.81; Breusch–Pagan p 0.053)	FPG	5.766	0.797; p < 0.001	1.823; p 0.002	2.819 to 8.608	2.05
Model 3 (TSH) (n = 267; R^2 = 0.276; max Cook's D 0.81; Breusch–Pagan p 0.053)	Age	0.055	0.070; p 0.431	0.097; p 0.569	-0.146 to 0.212	1.01
Model 3 (TSH) (n = 267; R^2 = 0.276; max Cook's D 0.81; Breusch–Pagan p 0.053)	Female	0.598	1.204; p 0.620	1.194; p 0.616	-1.462 to 2.866	1.01
Model 3 (TSH) (n = 267; R^2 = 0.276; max Cook's D 0.81; Breusch–Pagan p 0.053)	BMI	0.065	0.114; p 0.567	0.153; p 0.669	-0.185 to 0.330	1.23

The untransformed-TSH specification showed heteroscedasticity and influential observations driven by the extreme TSH range; its TSH coefficients were not significant under HC3 standard errors, which motivated the ln TSH specification used in the main text. The symmetry-check model illustrates that reversing outcome and predictors in cross-sectional data yields associations in both directions and cannot demonstrate bidirectional effects.

Figure S1. Regression residual diagnostics for the untransformed-TSH specification of the three original models (residuals versus fitted values, top; normal Q–Q plots of standardized residuals, bottom).

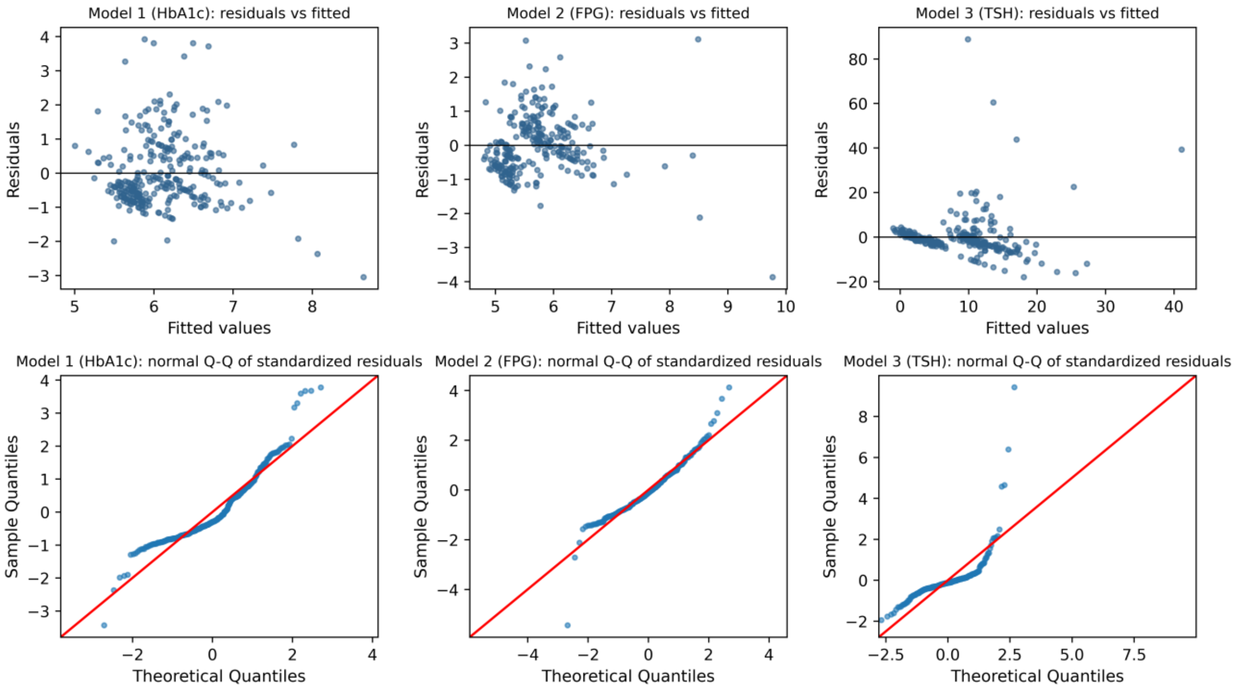

Figure S2. Sex-stratified scatter plots of TSH (logarithmic scale) against HbA1c in women and men, restricted to euthyroid and hypothyroid-pattern participants; each point is one participant.

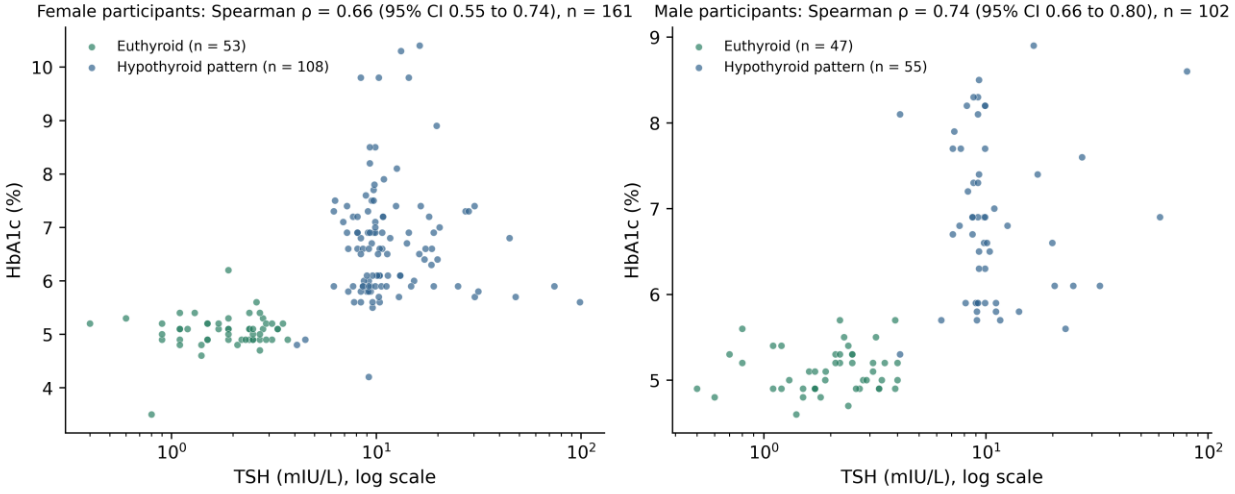

Analyses were performed in Python 3.12 (SciPy 1.17; statsmodels 0.15).
